# Influenza epidemiology and influenza vaccine effectiveness during the 2016–2017 season in the Global Influenza Hospital Surveillance Network (GIHSN)

**DOI:** 10.1186/s12889-019-6713-5

**Published:** 2019-05-02

**Authors:** Víctor Baselga-Moreno, Svetlana Trushakova, Shelly McNeil, Anna Sominina, Marta C. Nunes, Anca Draganescu, Serhat Unal, Parvaiz Koul, Jan Kyncl, Tao Zhang, Ainagul Kuatbayeva, Afif Ben-Salah, Elena Burtseva, Joan Puig-Barberà, Javier Díez-Domingo, for the Global Influenza Hospital Surveillance Network (GIHSN), B. Escribano-López, S. García Esteban, B. Guglieri-López, M. Martín-Navarro, A. Mira-Iglesias, M. J. Sánchez-Catalán, X. López-Labrador, E. Adriana-Magos, M. Carballido-Fernández, J. Mollar Maseres, M. Roldán-Aguado, J. Fernández-Dopazo, M. Tortajada-Girbés, P. Llorente-Nieto, G. Schwarz-Chavarri, E. Garina, L. Kisteneva, L. Kolobukhina, K. Krasnoslobotsev, I. Kruzhkova, L. Merkulova, E. Mukasheva, A. Ambrose, M. Andrew, M. ElSherif, D. MacKinnon-Cameron, M. Nichols-Evans, P. Ye, O. Afanasieva, A. Afanasieva, S. Demina, E. Dondurei, M. Eropkin, A. Fadeev, L. Generalova, A. Go, E. Golovacheva, V. Gonchar, A. Komissarov, N. Konovalova, S. Kuvarzina, T. Levanyuk, T. Lobova, L. Osidak, M. Pisareva, E. Rozhkova, K. Sintsova, Z. Sirotkina, E. Smorodintseva, K. Stolyarov, V. Sukhovetskaya, M. Tamila, L. Voloshuk, M. Yanina, P. Zarishnyuk, S. A. Madhi, V. Aramă, D. Florea, M. Luminos, D. Otelea, O. Sandulescu, O. Vlaicu, D. Pitigoi, K. Aykac, T. Bagcı Bosi, E. Bilgin, M. Durusu, A. Kara, L. Ozisik, S. Tanir Basaranoglu, T. Bedir Demirdag, O. Guzel Tunccan, O. Ozgen, H. Tezer, B. Gulhan, A. Ozkaya-Parlakay, M. Ozsoy, N. Tulek, M. Akcay Ciblak, A. Galindo Fraga, M. L. Guerrero Almeida, G. M. Ruiz-Palacios, A. de Colsa Ranero, W. Dolores Domínguez-Viveros, I. Jiménez-Escobar, J. P. Ramírez-Hinojosa, R. P. Vidal-Vázquez, D. de la Rosa-Zamboni, A. E. Gamiño-Arroyo, S. Moreno-Espinosa, A. Hernández, S. Ali, M. Khan, H. Mir Soumya, R. Yusuf, N. Bali, M. Havlickova, H. Jirincova, R. Kralova, Z. Mandakova, J. Prochazkova, H. Sebestova, D. Dvorska, K. Herrmanova, H. Rohacova, T. Rudova, I. Standerova, K. Chen, W. Shan, F. Zhang, G. Zhao, Y. Yan, J. Zheng, J. Pan, N. Gaukhar, S. Amine, J. Ben Khelil, M. Ben Jeema, M. Koubâa, K. Menif, A. Boukthir, S. Chlif, M. K. Dellagi, A. Gharbi, H. Louzir, R. Yazidi, W. Zid, A. Laguna, J. Pérez-Bao, N. Reyes, D. Coulibaly

**Affiliations:** 1 Fundación para el Fomento de la Investigación Sanitaria y Biomédica de la Comunidad Valenciana (FISABIO), 21 Cataluña Av, 46020 Valencia, Spain; 2 Ivanovsky Institute of Virology FSBI “N.F. Gamaleya FRCEM” Ministry of Health, Moscow, Russian Federation; 3 Canadian Center for Vaccinology, IWK Health Centre and Nova Scotia Health Authority, Halifax, Canada; 4 Research Institute of Influenza, WHO National Influenza Centre of Russia, St. Petersburg, Russian Federation; 5 Medical Research Council, Respiratory and Meningeal Pathogens Research Unit, University of the Witwatersrand, Johannesburg, South Africa; 6 Department of Science and Technology/National Research Foundation, Vaccine Preventable Diseases, University of the Witwatersrand, Johannesburg, South Africa; 7 National Institute of Infectious Diseases “Prof. Dr. Matei Bals”, Bucharest (INBI “Prof. Dr. Matei Bals”), București, Romania; 8 Turkish Society of Internal Medicine, Ankara, Turkey; 9 Department of Internal and Pulmonary Medicine, Sher-i-Kashmir Institute of Medical Sciences (SKIMS), Soura, India; 10 National Institute of Public Health, Prague, Czech Republic; 11 Fudan University, Shanghai, China; 12 Center for Sanitary-Epidemiological Expertise and Monitoring, Almaty, Kazakhstan; 13 Pasteur Institute of Tunis, Tunis, Tunisia; 14 College of Medicine and Medical Sciences, Manama, Bahrain

**Keywords:** Influenza virus, Surveillance, Vaccine effectiveness, Epidemiology

## Abstract

**Background:**

The Global Influenza Hospital Surveillance Network (GIHSN) aims to determine the burden of severe influenza disease and Influenza Vaccine Effectiveness (IVE). This is a prospective, active surveillance and hospital-based epidemiological study to collect epidemiological data in the GIHSN. In the 2016–2017 influenza season, 15 sites in 14 countries participated in the GIHSN, although the analyses could not be performed in 2 sites. A common core protocol was used in order to make results comparable. Here we present the results of the GIHSN 2016–2017 influenza season.

**Methods:**

A RT-PCR test was performed to all patients that accomplished the requirements detailed on a common core protocol. Patients admitted were included in the study after signing the informed consent, if they were residents, not institutionalised, not discharged in the previous 30 days from other hospitalisation with symptoms onset within the 7 days prior to admission. Patients 5 years old or more must also complied the Influenza-Like Illness definition. A test negative-design was implemented to perform IVE analysis. IVE was estimated using a logistic regression model, with the formula IVE = (1-aOR) × 100, where aOR is the adjusted Odds Ratio comparing cases and controls.

**Results:**

Among 21,967 screened patients, 10,140 (46.16%) were included, as they accomplished the inclusion criteria, and tested, and therefore 11,827 (53.84%) patients were excluded. Around 60% of all patients included with laboratory results were recruited at 3 sites. The predominant strain was A(H3N2), detected in 63.6% of the cases (1840 patients), followed by B/Victoria, in 21.3% of the cases (618 patients). There were 2895 influenza positive patients (28.6% of the included patients). A(H1N1)pdm09 strain was mainly found in Mexico. IVE could only be performed in 6 sites separately. Overall IVE was 27.24 (95% CI 15.62–37.27. Vaccination seemed to confer better protection against influenza B and in people 2–4 years, or 85 years old or older. The aOR for hospitalized and testing positive for influenza was 3.02 (95% CI 1.59–5.76) comparing pregnant with non-pregnant women.

**Conclusions:**

Vaccination prevented around 1 in 4 hospitalisations with influenza. Sparse numbers didn’t allow estimating IVE in all sites separately. Pregnancy was found a risk factor for influenza, having 3 times more risk of being admitted with influenza for pregnant women.

**Electronic supplementary material:**

The online version of this article (10.1186/s12889-019-6713-5) contains supplementary material, which is available to authorized users.

## Background

Influenza is a major public health problem that can cause hospitalisations, and it is related with respiratory failures [1, 2]. The Global Influenza Hospital Surveillance Network (GIHSN) is an international public-private collaboration that started in 2012. The GIHSN goals are to improve understanding of influenza epidemiology, quantifying the circulation of the different types and subtypes of influenza, in order to measure the effectiveness of seasonal influenza vaccines and better inform public health policy decisions. We conduct a prospective, active surveillance, hospital-based epidemiological study that collects epidemiological and virological data from those sites that are included in the network. Each season results are presented in annual meetings and, since 2012, have been published [3–6], with the agreement of the Principal Investigators of all concerned sites. The implementation and data collection for the last season (2016–2017) was led by the Foundation for the Promotion of Health and Biomedical Research of Valencia Region (FISABIO), a regional public health institution in Valencia, Spain, and funded by the Foundation for Influenza Epidemiology. Fifteen sites in fourteen countries participated in the GIHSN in the season 2016–2017. Among them, there were 12 sites (St. Petersburg, Moscow, Kazakhstan, Czech Rep., Canada, Romania, Turkey, Spain, Tunisia, Suzhou/Shanghai, India and Mexico) from Northern Hemisphere countries not situated in the tropics and three sites (Ivory Coast, Peru and South Africa) from the tropics or the Southern Hemisphere. Since Peru and Ivory Coast only reported two positive cases for influenza in the influenza season, the analysis was performed without the data from these countries, and therefore, results are reported for 13 sites. A common core protocol and standard operating procedures are used for all participating sites, in order to allow comparisons among countries, and analyse results of all sites.

## Methods

**Fig. 1:**
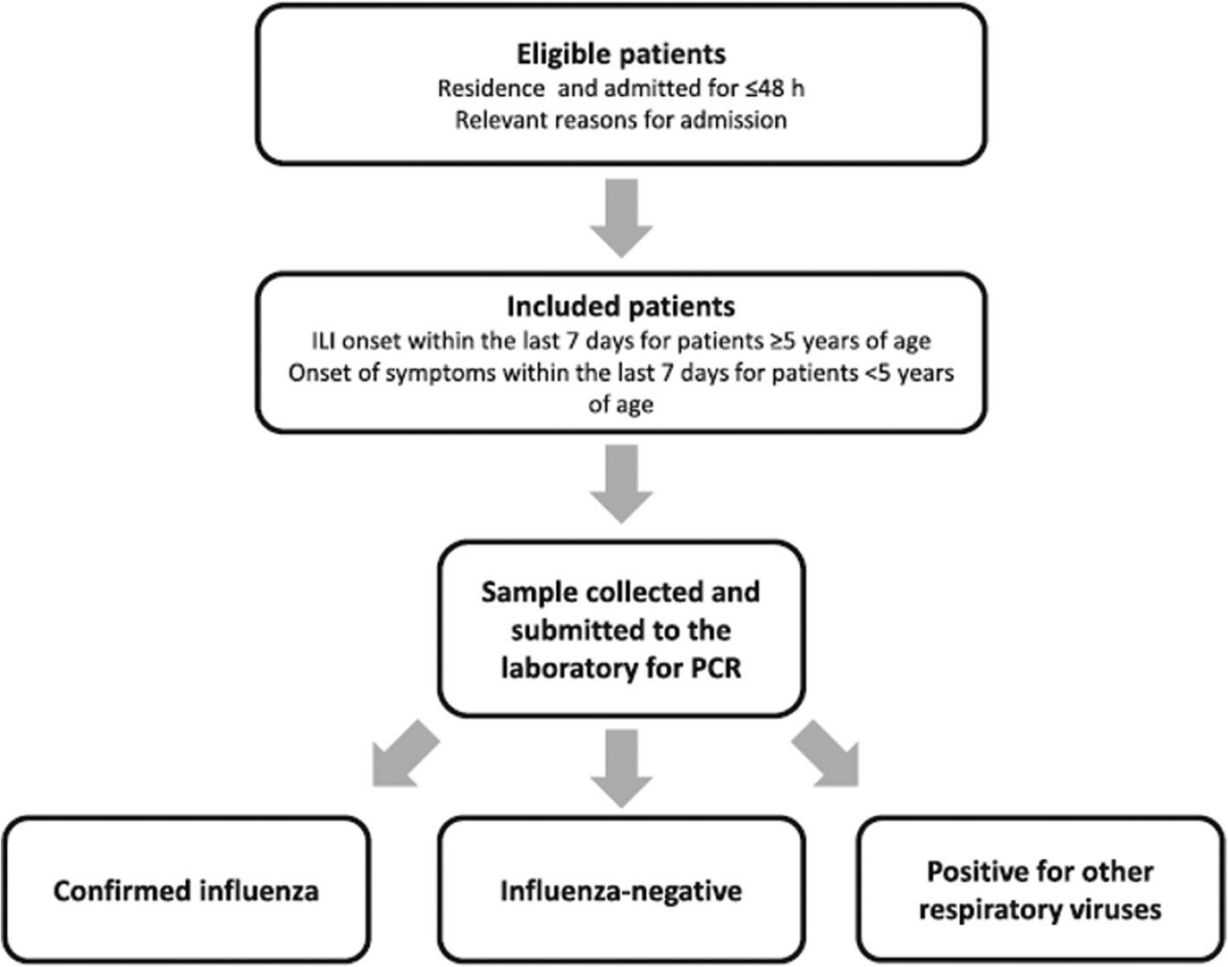
Overview of the methodology used by the GIHSN

This study aims to determine the frequency of influenza-related hospitalisations in different countries, by circulating strains and age groups, to study risk factors for influenza-associated hospitalisations and estimate Influenza Vaccine Effectiveness (IVE) by site, age group and strain. Each site had one or more hospitals that recruited patients for the study, between October 2016 and May 2017 in Northern Hemisphere sites, except China, whose patients were recruited between June and September. For Southern Hemisphere sites, patients were recruited between May and November. Patients were included in the study if they presented any of the admission diagnoses included in the protocol, and only if they signed the informed consent to participate in the study. Among them, we selected for the study only those who were residents in the predefined hospital catchment’s area in the previous past 6 months, who were not institutionalised, who hadn’t been discharged from other hospitalisation in the last 30 days, and who had symptoms possibly related to influenza in 7 days or less prior to admission (Fig. 1). We also excluded patients who had previously tested positive for influenza in the current season, and also patients for whom the difference between the date of the onset of symptoms and the date of swabbing was 10 days or more (that is, those admitted after the 7th day after the onset of symptoms+maximum delay in swabbing). For patients 5 years old or more, they must also have complied with the Influenza-Like Illness (ILI) definition, detailed in European Centre for Disease Prevention and Control (ECDC) protocols, according to the decision of the Commission of the European Union of 8 August 2012 [7]. Patients enrolled outside the influenza epidemic period of each of the participating sites were also excluded. Influenza seasons were previously determined by each site, following recommendations of previous studies [8]. This methodology has been used in the GIHSN since the beginning of the network, and has been previously described [9]. For patients under 14 years old, nasal and/or nasopharyngeal swabs were collected, whereas, for patients 14 years old or more, pharyngeal and/or nasopharyngeal swabs were taken. Reverse transcription-polymerase chain reaction (RT-PCR) was used, according to each site’s protocol, in order to detect influenza virus; viral subtyping was performed in order to identify A(H1N1)pdm09, A(H3N2), B/Yamagata-lineage, and B/Victoria-lineage strains in the positive specimens.

We performed a test-negative study [10] in order to compare positives (cases) and negatives (controls) for influenza and estimate Influenza Vaccine Effectiveness (IVE). Odds Ratios were used to estimate IVE, comparing cases and controls of patients depending on the vaccination status. Patients were considered vaccinated if they received an influenza vaccine in the current season, at least 15 days before the onset of symptoms. Patients with contra-indication to influenza vaccination were excluded from the IVE analysis, but were included in the analysis regarding influenza circulation. Vaccination status was ascertained either by recall or by vaccination registries. Adjusted odds ratios (aOR) were calculated using a logistic regression model including sex, occupational social class, obesity status, pregnancy, underlying conditions, general practitioner (GP) consultations in last 3 months, smoking habits, days from onset of symptoms to swabbing as fixed effects, age and epidemiological week of admission using cubic splines, and site as a cluster variable, in order to consider sites variability [11]. IVE was calculated as (1-aOR) × 100. The same factors were used to adjust IVE by strain or age group. The variables relative to the Barthel Index (in patients 65 years old or older) and the previous hospitalisations in the last year were initially considered to be included in the model, but were excluded from the final model as they were not statistically significant considering all variables mentioned above. The model did not include the number of consultations at the GP in the last 3 months to estimate IVE in Canada, as this site did not provide information for this variable. Severe outcomes were also studied, defining them as an influenza positive patient admitted to ICU during the hospitalisation, or with COPD exacerbation, respiratory failure, any cardiovascular complication, shock or death during hospitalisation. Heterogeneity was studied, using the I^2^ test, and considering that heterogeneity was relevant if I^2^ ≥ 50% [12, 13].

## Results

### Included patients: distribution, characteristics and influenza positives and negatives

**Fig. 2:**
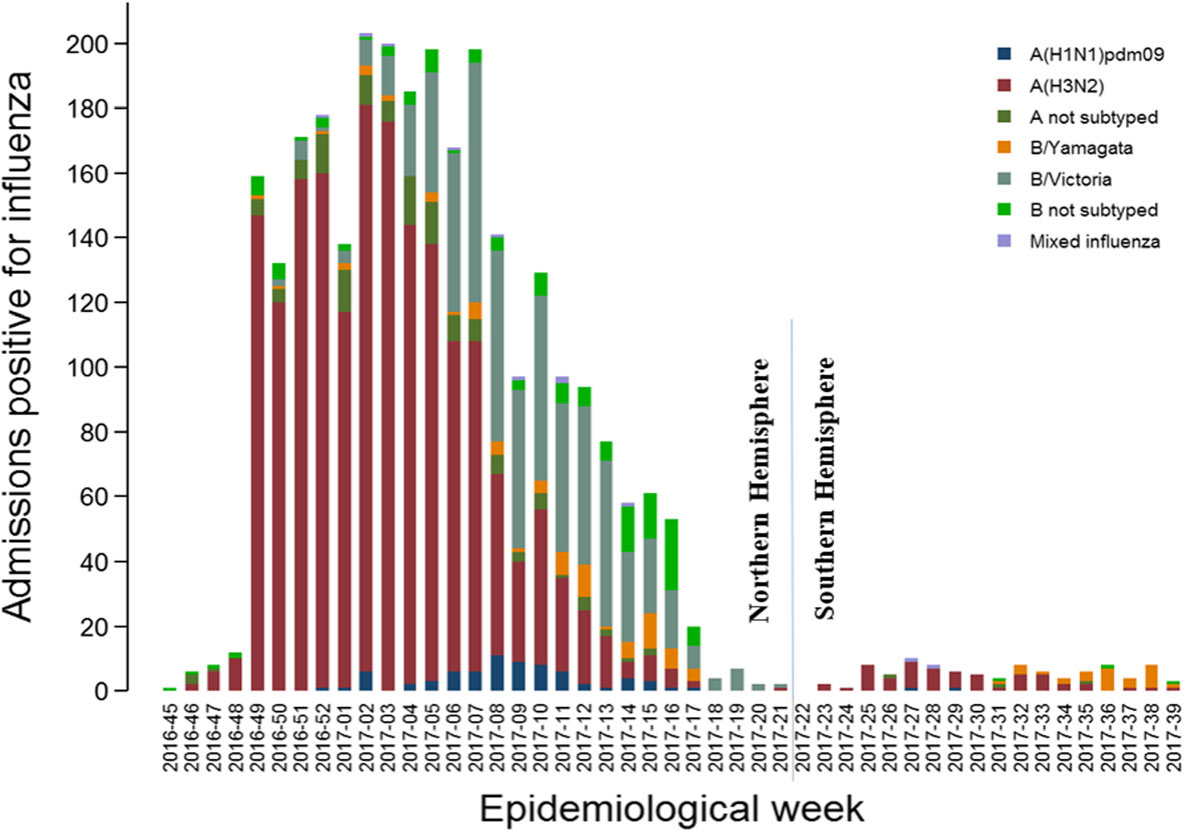
Influenza-associated admissions by epidemiological week and virus type/subtype

There were 21,967 eligible admissions between October 1, 2016 and November 9, 2017. However, only 10,140 patients complied with the conditions described above, and had laboratory results, hence only these were included in the analysis. Among them, 2895 (28.6%) tested positive for influenza, and 7245 (71.4%) tested negative for influenza (Table 1). The most common reason of exclusion was the fact that patients didn’t have ILI symptoms in the 7 days previous to admission. It is important to note that 2/3 of all included patients in the GIHSN came from 4 sites (St. Petersburg, Moscow, Canada and Valencia). These 4 sites also have the highest numbers of influenza positive cases, including 77.8% of all influenza positives in the GIHSN, and 84.3% of the A(H3N2) influenza positives among all participant sites. A (H3N2) was the predominant strain this season, being detected in 63.6% of all influenza positive cases (1840 patients), followed by B/Victoria, with 21.3% among the influenza positive cases (618 patients) (Table 1). Influenza A(H3N2) was detected throughout the season, whereas B/Victoria started to increase in the second week of 2017 in the Northern Hemisphere, and in the 31st week of 2017 in the Southern Hemisphere, approximately in the middle of the season in each Hemisphere (Fig. 2).

**Table 1:** Patients included and excluded in the current analyses, inclusion criteria and influenza laboratory results

Category	St. Pet		Moscow		Kazakhstan		Czech Rep.		Canada		Romania		Turkey		Valencia		Tunisia		Suzhou/ Shanghai		India		Mexico		South Africa		Total	
	n	%	n	%	n	%	n	%	n	%	N	%	n	%	n	%	n	%	n	%	n	%	n	%	n	%	n	%
Screened admissions	2012		2244		661		201		2450		902		917		6913		106		1264		693		1480		2124		21967	
Exclusion criteria																												
Non resident	2	0.1	167	7.4	0	0.0	3	1.5	1	0.0	394	43.7	78	8.5	25	0.4	9	8.5	180	14.2	5	0.7	294	19.9	0	0.0	1158	5.3
institutionalised	1	0.0	19	0.8	21	3.2	0	0.0	461	18.8	1	0.1	20	2.2	358	5.2	0	0.0	1	0.1	0	0.0	9	0.6	0	0.0	891	4.1
Previous discharged < 30 days	3	0.1	114	5.1	44	6.7	7	3.5	145	5.9	68	7.5	173	18.9	1131	16.4	5	4.7	65	5.1	33	4.8	216	14.6	0	0.0	2004	9.1
Unable to communicate	10	0.5	136	6.1	0	0.0	11	5.5	0	0.0	0	0.0	50	5.5	367	5.3	0	0.0	30	2.4	0	0.0	126	8.5	282	13.3	1012	4.6
Not giving consent	44	2.2	8	0.4	49	7.4	13	6.5	0	0.0	1	0.1	15	1.6	275	4.0	0	0.0	3	0.2	1	0.1	54	3.6	90	4.2	553	2.5
No ILI symptoms ≥5 years	0	0.0	42	1.9	9	1.4	37	18.4	573	23.4	41	4.5	140	15.3	2164	31.3	0	0.0	0	0.0	0	0.0	108	7.3	215	10.1	3329	15.2
Admission within 7 days of symptoms onset	4	0.2	124	5.5	279	42.2	8	4.0	137	5.6	4	0.4	3	0.3	335	4.8	4	3.8	301	23.8	2	0.3	216	14.6	170	8.0	1587	7.2
Previous influenza infection	2	0.1	0	0.0	0	0.0	0	0.0	0	0.0	6	0.7	7	0.8	1	0.0	0	0.0	15	1.2	0	0.0	9	0.6	1	0.0	41	0.2
Onset of symptoms to swab > 9 days	0	0.0	1	0.0	0	0.0	0	0.0	0	0.0	0	0.0	2	0.2	1	0.0	6	5.7	1	0.1	0	0.0	0	0.0	0	0.0	11	0.1
Sample inadequate	0	0.0	0	0.0	0	0.0	0	0.0	0	0.0	0	0.0	0	0.0	0	0.0	0	0.0	0	0.0	0	0.0	0	0.0	0	0.0	0	0.0
Sample lost	0	0.0	0	0.0	0	0.0	0	0.0	0	0.0	0	0.0	0	0.0	0	0.0	25	23.6	0	0.0	0	0.0	0	0.0	0	0.0	25	0.1
Recruited outside periods with continuous influenza positive admissions	9	0.4	13	0.6	100	15.1	11	5.5	1	0.0	0	0.0	16	1.7	131	1.9	18	17.0	198	15.7	159	22.9	98	6.6	462	21.8	1216	5.5
Included with valid laboratory results	1937	96.3	1620	72.2	159	24.1	111	55.2	1132	46.2	387	42.9	413	45.0	2125	30.7	39	36.8	470	37.2	493	71.1	350	23.6	904	42.6	10140	46.2
RT-PCR result																												
Influenza negative	1417	73.2	869	53.6	128	80.5	69	62.2	414	36.6	221	57.1	311	75.3	1862	87.6	30	76.9	433	92.1	425	86.2	259	74.0	807	89.3	7245	71.4
Influenza positive	520	26.8	751	46.4	31	19.5	42	37.8	718	63.4	166	42.9	102	24.7	263	12.4	9	23.1	37	7.9	68	13.8	91	26.0	97	10.7	2895	28.6
Subtype and lineage																												
A(H1N1)pdm09	1	0.2	0	0.0	0	0.0	1	2.4	3	0.3	0	0.0	0	0.0	0	0.0	1	11.1	1	2.7	11	16.2	56	61.5	2	2.1	76	2.6
A(H3N2)	296	56.9	420	55.9	15	48.4	32	76.2	585	51.7	39	23.5	81	79.4	251	95.4	6	66.7	21	56.8	21	30.9	12	13.2	61	62.9	1840	63.6
A not subtyped	34	6.5	4	0.5	0	0.0	2	4.8	67	5.9	4	2.4	3	2.9	12	4.6	0	0.0	0	0.0	0	0.0	0	0.0	3	3.1	129	4.5
B/Yamagata lineage	2	0.4	0	0.0	0	0.0	4	9.5	35	3.1	0	0.0	19	18.6	0	0.0	2	22.2	1	2.7	0	0.0	15	16.5	30	30.9	108	3.7
B/Victoria lineage	187	36.0	299	39.8	0	0.0	1	2.4	4	0.4	74	44.6	2	2.0	0	0.0	0	0.0	14	37.8	37	54.4	0	0.0	0	0.0	618	21.3
B not subtyped	0	0.0	28	3.7	16	51.6	2	4.8	24	2.1	50	30.1	1	1.0	0	0.0	0	0.0	0	0.0	0	0.0	11	12.1	3	3.1	135	4.7

In the Northern Hemisphere, there was a significant increase in the number of influenza cases in week #49 of 2016, with a peak in the number of positive cases during the second week of 2017 and starting to descend at the eighth week of 2017. Influenza B/Victoria started to increase clearly in the second week of 2017, as A(H3N2) started to descend. 70.3% of all influenza cases were positive for influenza A, whereas 29.7% were positive for influenza B, with a clear different distribution among sites.

**Fig. 3:**
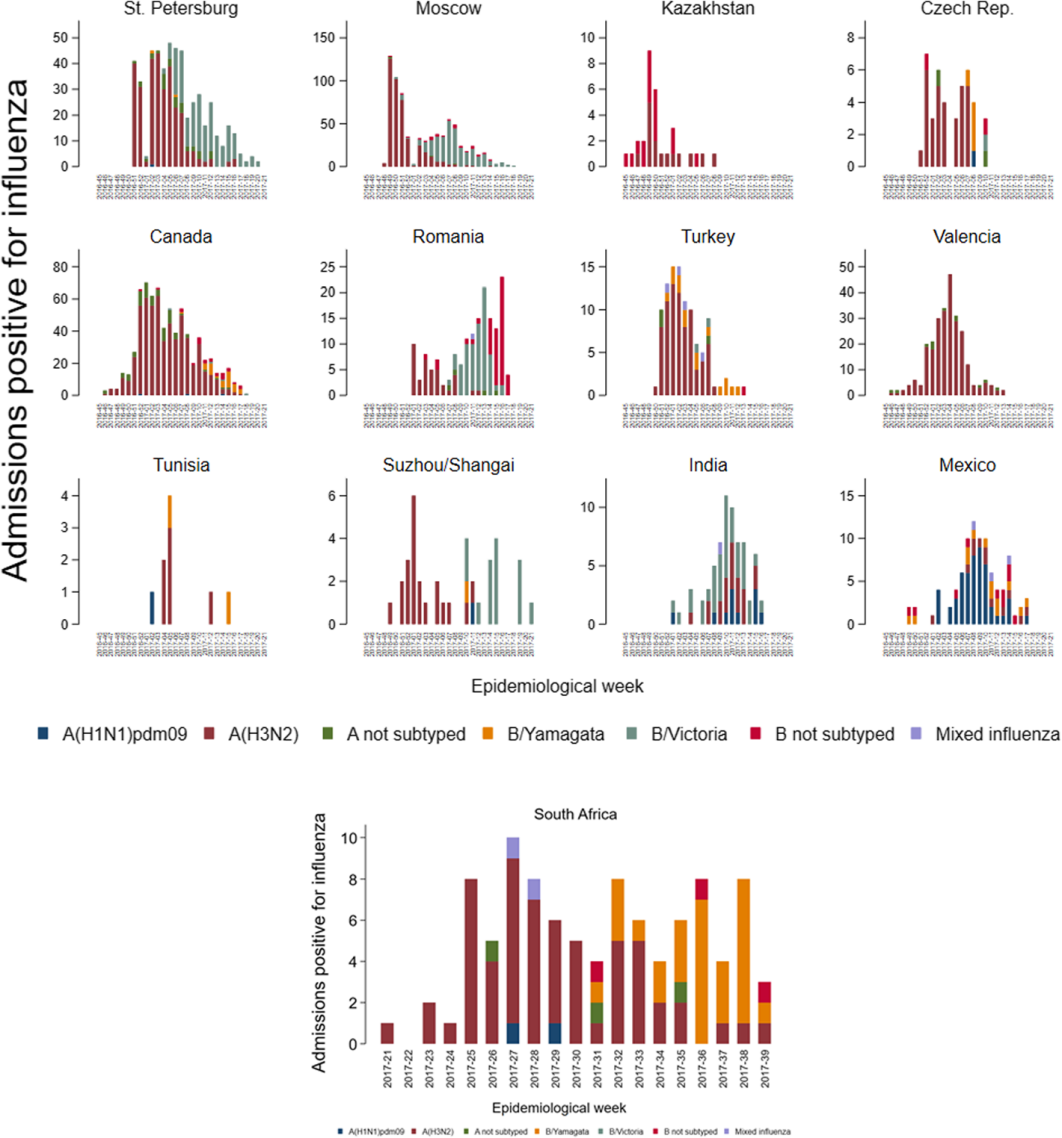
Admissions with influenza by site, epidemiological week and virus type/subtype

A(H3N2) was predominant in all sites, except in Mexico, where the predominant strain was A(H1N1)pdm09, and Romania and India with a predominance of B/Victoria-lineage. Both B lineages circulated during this season, with geographical differences, so in Canada, Czech Republic, Turkey, Tunisia, Mexico and South Africa, B/Yamagata was more often detected, while the B/Victoria was elsewhere. Influenza B cases generally appeared as a second influenza wave (Fig. 3). In Valencia, no cases were positive for influenza B.

**Fig. 4:**
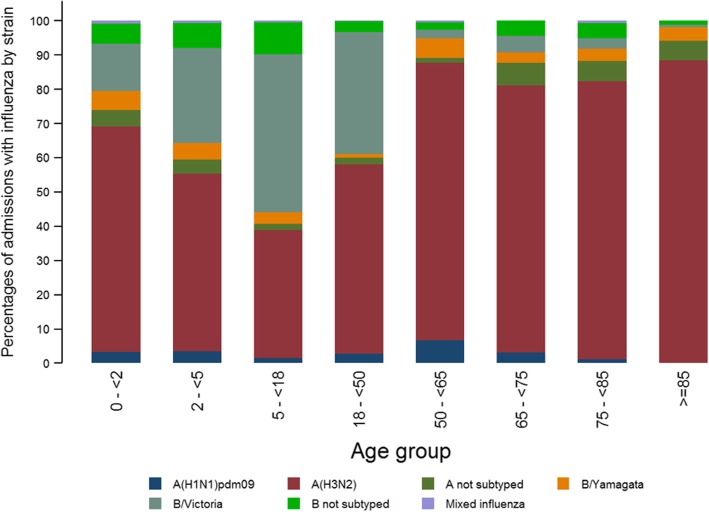
Percentages of influenza-associated admissions by age group and type/subtype

Influenza B was mainly observed in the youngest, and was the predominant strain in the age group 5–17 years old. Among the two influenza B lineages, in general B/Victoria was detected more often than B/Yamagata, except in the age group 50–64 years (Fig. 4).

**Fig. 5:**
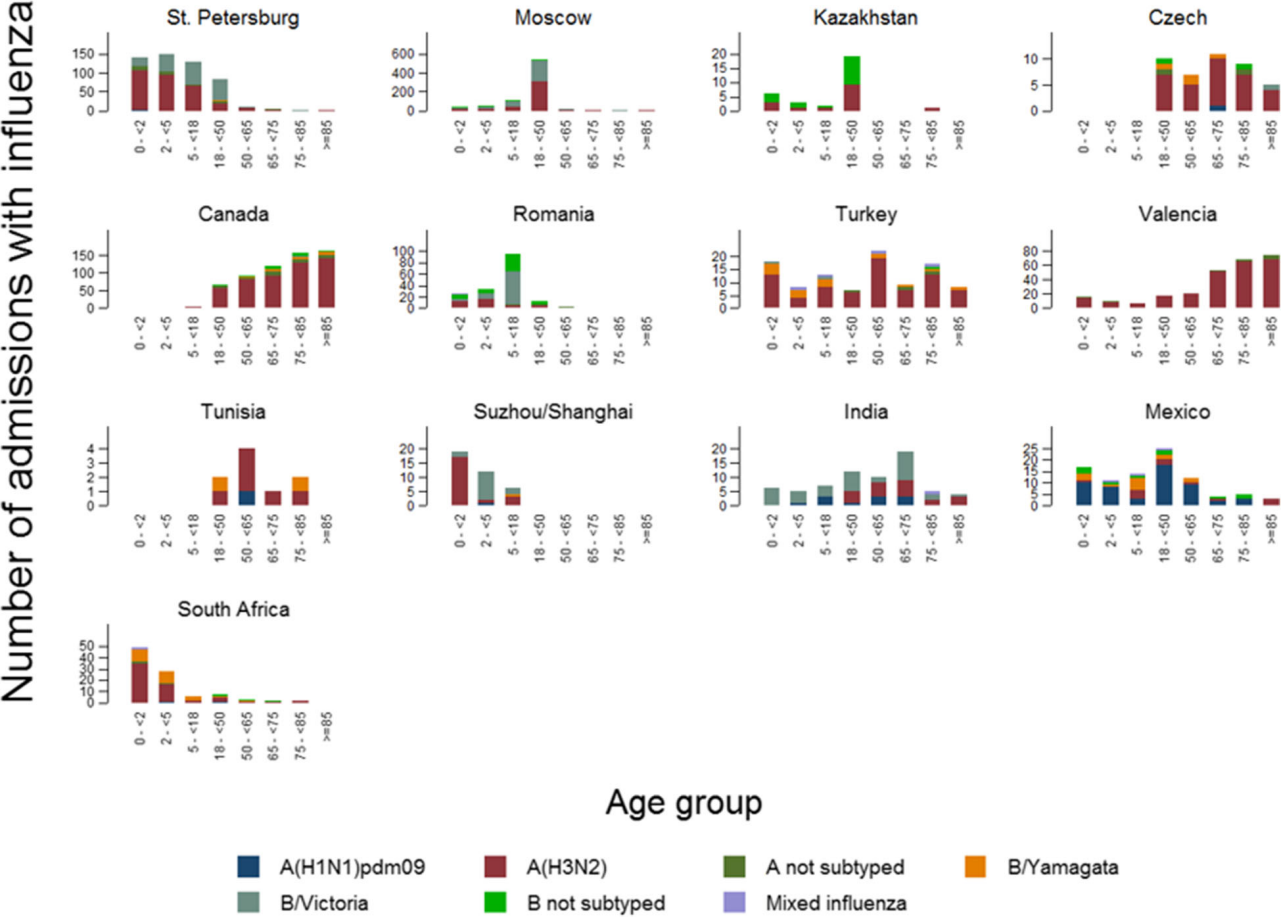
Admissions with influenza by site, age group and virus type/subtype

The distribution of influenza cases among the age groups was clearly different among sites, but differences were mainly due to the characteristics of the participating hospitals for each site. Tunisia and Czech Republic only recruited patients 18 years old or older, while Suzhou/Shanghai only enrolled patients under 18 years old. In Moscow, the majority of influenza positives were pregnant women (which represented the 49.4% of the included patients), and therefore, the highest number of influenza positives among the different age groups was situated in the age group 18–49 years old in this site. Influenza positive cases were mainly found in patients 65 years old or older in Valencia and Canada, but 89.8% of the included patients from Canada were 50 years old or older. In St. Petersburg and South Africa, due to the characteristics of the patients of the participating hospitals (mainly children) there were more influenza positive cases in the youngest groups (Fig. 5).

25.8% of the included patients were previously hospitalised in the same year and 36.6% of the included patients had at least one underlying condition, but this percentage varied among sites, in Canada, for example, more than 90% of the included patients had at least one underlying condition, whereas in St. Petersburg, this percentage was lower than 10% and in Turkey was 48.2%, but these percentages could be related to the age distribution of the included patients in each site. Among the different comorbidities, the most common were cardiovascular (20.7% of the included patients), diabetes (10.4%) and chronic obstructive pulmonary disease (COPD) (9.9%). Obesity was also found in more than 14% of the included patients, being more relevant in Canada (29.6%), Valencia (26.3%) and Czech Republic (23.4%). Moscow was the site with the highest number of pregnant women among all sites (800 pregnant in Moscow among 940 pregnant women in all sites), being 49.4% of the included patients in this site. In Kazakhstan, pregnant women represented 22.6% of the included patients. The Barthel Index in those over 65 years showed that almost 90% of these subjects were not dependent or had a mild dependence. 68.3% of the patients who tested negative for influenza were swabbed from 0 to 4 days after symptoms started, but this percentage was 78.4% for influenza positive cases (*p*-value< 0.0001).

Vaccination coverage differed among sites. Patients were considered as vaccinated if vaccination was at least 15 days before symptoms onset (Table 2). Targeted patients for vaccination criteria were different among sites (Additional file 1: Complementary Table S1). Vaccination coverage was 11.1% among the influenza positives and 18.4% among the influenza negatives overall. Cardiovascular diseases, renal impairment, chronic obstructive pulmonary disease and diabetes were the most common comorbidities among influenza positives (Table 3). Seasonality had also a clear geographical distribution. Sites in higher latitudes had, generally, an earlier start of the influenza season.

**Table 2:** Characteristics of included patients overall and by site

Characteristic	St. Pet		Moscow		Kazakhstan		Czech Rep.		Canada		Romania		Turkey		Valencia		Tunisia		Suzhou/ Shanghai		India		Mexico		South Africa		Total	
	*N* = 1937		*N* = 1620		*N* = 159		*N* = 111		*N* = 1132		*N* = 387		*N* = 413		*N* = 2125		*N* = 39		*N* = 470		*N* = 493		*N* = 350		*N* = 904		*N* = 10,140	
	n	%	n	%	n	%	n	%	n	%	n	%	n	%	n	%	n	%	n	%	n	%	n	%	n	%	n	%
Age in years, median (range)	3 (0–87)		24 (0–91)		17 (1–76)		64 (18–90)		76 (17–105)		5 (0–63)		3 (0–95)		68 (0–102)		58 (14–84)		0 (0–13)		60 (0–99)		3 (0–96)		0 (0–91)		20 (0–105)	
Age group																												
0–1 y	684	35.3	167	10.3	34	21.4	0	0.0	0	0.0	89	23.0	179	43.3	421	19.8	0	0.0	334	71.1	57	11.6	151	43.1	576	63.7	2692	27.0
2–4 y	483	24.9	156	9.6	33	20.8	0	0.0	0	0.0	87	22.5	39	9.4	108	5.1	0	0.0	96	20.4	19	3.9	50	14.3	146	16.2	1217	12.2
5–17 y	310	16.0	182	11.2	14	8.8	0	0.0	1	0.1	118	30.5	32	7.7	54	2.5	1	2.6	40	8.5	16	3.2	43	12.3	16	1.8	827	8.3
18–49 y	388	20.0	1052	64.9	73	45.9	37	33.3	97	8.6	72	18.6	14	3.4	145	6.8	9	23.1	0	0.0	79	16.0	52	14.9	82	9.1	2100	21.1
50–64 y	49	2.5	34	2.1	2	1.3	20	18.0	156	13.8	21	5.4	45	10.9	227	10.7	12	30.8	0	0.0	100	20.3	21	6.0	48	5.3	735	7.4
65–74 y	12	0.6	12	0.7	2	1.3	24	21.6	196	17.3	0	0.0	29	7.0	335	15.8	8	20.5	0	0.0	143	29.0	11	3.1	21	2.3	793	8.0
75–84 y	9	0.5	10	0.6	1	0.6	20	18.0	264	23.3	0	0.0	55	13.3	462	21.7	9	23.1	0	0.0	51	10.3	11	3.1	11	1.2	903	9.0
≥ 85 y	2	0.1	7	0.4	0	0.0	10	9.0	246	21.7	0	0.0	20	4.8	373	17.6	0	0.0	0	0.0	28	5.7	11	3.1	4	0.4	701	7.0
Sex																												
Male	1050	54.2	607	37.5	76	47.8	64	57.7	541	47.8	205	53.0	224	54.2	1125	52.9	27	69.2	287	61.1	242	49.1	171	48.9	486	53.8	5105	50.3
Female	887	45.8	1013	62.5	83	52.2	47	42.3	591	52.2	182	47.0	189	45.8	1000	47.1	12	30.8	183	38.9	251	50.9	179	51.1	418	46.2	5035	49.7
Chronic conditions																												
0	1758	90.8	1382	85.3	111	69.8	35	31.5	99	8.7	349	90.2	214	51.8	803	37.8	7	17.9	443	94.3	129	26.2	218	62.3	878	97.1	6426	63.4
1	157	8.1	187	11.5	42	26.4	40	36.0	307	27.1	28	7.2	87	21.1	626	29.5	18	46.2	27	5.7	182	36.9	85	24.3	26	2.9	1812	17.9
≥2	22	1.1	51	3.1	6	3.8	36	32.4	726	64.1	10	2.6	112	27.1	696	32.8	14	35.9	0	0.0	182	36.9	47	13.4	0	0.0	1902	18.7
Previously hospitalised (last 12 months)																												
No	1447	74.7	1354	83.6	143	89.9	80	72.1	–	–	279	72.1	272	65.9	1457	68.6	30	76.9	329	70.0	312	63.3	240	68.6	745	82.4	6688	74.2
Yes	490	25.3	266	16.4	16	10.1	31	27.9	–	–	108	27.9	141	34.1	668	31.4	9	23.1	141	30.0	181	36.7	110	31.4	159	17.6	2320	25.8
Underlying chronic conditions																												
Cardiovascular disease	49	2.5	70	4.3	5	3.1	50	45.0	872	77.0	17	4.4	110	26.6	602	28.3	15	38.5	24	5.1	199	40.4	65	18.6	16	1.8	2094	20.7
Chronic obstructive pulmonary disease	21	1.1	23	1.4	24	15.1	7	6.3	134	11.8	1	0.3	70	16.9	500	23.5	21	53.8	0	0.0	177	35.9	28	8.0	2	0.2	1008	9.9
Asthma	28	1.4	29	1.8	0	0.0	7	6.3	146	12.9	2	0.5	46	11.1	162	7.6	2	5.1	2	0.4	5	1.0	27	7.7	7	0.8	463	4.6
Immunodeficiency/organ transplant	13	0.7	1	0.1	1	0.6	4	3.6	114	10.1	8	2.1	18	4.4	29	1.4	1	2.6	0	0.0	17	3.4	16	4.6	0	0.0	222	2.2
Diabetes	7	0.4	16	1.0	3	1.9	25	22.5	344	30.4	6	1.6	47	11.4	500	23.5	7	17.9	0	0.0	71	14.4	23	6.6	0	0.0	1049	10.3
Renal impairment	4	0.2	74	4.6	15	9.4	3	2.7	167	14.8	4	1.0	27	6.5	274	12.9	4	10.3	1	0.2	29	5.9	14	4.0	1	0.1	617	6.1
Neuromuscular disease	56	2.9	29	1.8	6	3.8	6	5.4	182	16.1	0	0.0	31	7.5	57	2.7	1	2.6	0	0.0	45	9.1	13	3.7	0	0.0	426	4.2
Neoplasm	0	0.0	15	0.9	0	0.0	11	9.9	239	21.1	5	1.3	27	6.5	141	6.6	0	0.0	0	0.0	33	6.7	8	2.3	0	0.0	479	4.7
Cirrhosis/liver disease	18	0.9	18	1.1	1	0.6	3	2.7	22	1.9	5	1.3	6	1.5	62	2.9	0	0.0	0	0.0	0	0.0	0	0.0	0	0.0	135	1.3
Autoimmune disease	7	0.4	29	1.8	0	0.0	5	4.5	1	0.1	5	1.3	5	1.2	43	2.0	2	5.1	0	0.0	22	4.5	12	3.4	0	0.0	131	1.3
Pregnant (women 15–45 y)	72	3.7	800	49.4	36	22.6	3	2.7	14	1.2	7	1.8	0	0.0	2	0.1	0	0.0	0	0.0	0	0.0	2	0.6	4	0.4	940	9.3
Obese (all ages)	165	8.5	150	9.3	13	8.2	26	23.4	197	29.6	35	9.0	76	18.4	559	26.3	5	12.8	77	16.4	37	7.5	46	13.1	71	9.6	1457	14.4
Outpatient consultations last 3 months																												
0	894	46.2	658	40.6	116	73.0	33	29.7	–	–	166	42.9	148	35.8	233	11.0	14	35.9	44	9.4	120	24.3	81	23.1	776	85.8	3283	36.4
1	624	32.2	238	14.7	43	27.0	34	30.6	–	–	121	31.3	100	24.2	413	19.4	11	28.2	123	28.3	59	12.0	70	20.0	82	9.1	1928	21.4
≥ 2	419	21.6	724	44.7	0	0.0	44	39.6	–	–	100	25.8	165	40.0	1479	69.6	14	35.9	293	62.3	314	63.7	199	56.9	46	5.1	3797	42.2
Smoking habits (patients ≥18 y)																												
Never smoker	222	48.3	698	62.6	58	74.4	51	45.9	431	43.5	55	59.1	85	52.1	784	50.8	15	39.5	0	–	198	49.4	57	53.8	102	61.4	2756	52.4
Past smoker	46	10.0	263	23.6	16	20.5	24	21.6	387	39.1	6	6.5	59	36.2	464	30.1	12	31.6	0	–	121	30.2	34	32.1	35	21.1	1467	27.9
Current smoker	192	41.7	154	13.8	4	5.1	36	32.4	172	17.4	32	34.4	19	11.7	294	19.1	11	28.9	0	–	82	20.4	15	14.2	29	17.5	1040	19.7
Functional status impairment (Barthel score; patients ≥65 y)																												
Total (0–15)	0	0.0	0	0.0	0	0.0	0	0.0	14	2.8	0	–	8	8.3	94	8.0	0	0.0	0	–	13	5.9	0	0.0	1	5.6	130	6.0
Severe (20–35)	0	0.0	0	0.0	0	0.0	0	0.0	11	2.2	0	–	3	3.1	26	2.2	3	17.6	0	–	3	1.4	3	9.1	1	5.6	50	2.3
Moderate (40–55)	0	0.0	2	6.9	0	0.0	1	1.9	15	3.0	0	–	3	3.1	54	4.6	8	47.1	0	–	8	3.6	1	3.0	1	5.6	93	4.3
Mild (60–90)	4	18.2	7	24.1	2	66.7	14	25.9	90	17.9	0	–	35	36.5	261	22.3	4	23.5	0	–	62	27.9	12	36.4	9	50.0	500	23.1
Minimal (95–100)	18	81.8	20	69.0	1	33.3	39	72.2	373	74.2	0	–	47	49.0	735	62.8	2	11.8	0	–	136	61.3	17	51.5	6	33.3	1394	64.3
Sampling time																												
0–2 days	1160	59.9	843	52.0	109	68.6	31	27.9	474	41.9	76	19.6	59	14.3	386	18.2	7	17.9	8	1.7	44	8.9	67	19.1	321	39.1	3585	35.6
3–4 days	568	29.3	595	36.7	46	28.9	42	37.8	387	34.2	155	40.1	161	39.0	892	42.0	14	35.9	107	22.8	175	35.5	123	35.1	308	37.5	3573	35.5
5–7 days	209	10.8	179	11.0	4	2.5	37	33.3	259	22.9	144	37.2	181	43.8	655	30.8	18	46.2	264	56.2	274	55.6	141	40.3	140	17.1	2505	24.9
8–9 days	0	0.0	3	0.2	0	0.0	1	0.9	12	1.1	12	3.1	12	2.9	192	9.0	0	0.0	91	19.4	0	0.0	19	5.4	52	6.3	394	3.9
Influenza vaccination ≥15 days from symptom onset	86	4.4	65	4.0	0	0.0	6	5.4	139	12.3	7	1.8	21	5.1	825	38.8	2	5.1	1	0.2	11	2.2	49	14.0	5	0.6	1217	12.0
Influenza vaccination ≥15 days from symptom onset (age ≥ 65)	2	8.7	5	17.2	0	0.0	6	11.1	124	14.1	0	–	14	13.5	701	59.9	2	11.8	0	–	5	2.3	9	27.3	0	0.0	868	33.8
Influenza vaccination ≥15 days from symptom onset (targeted groups)	65	4.5	30	2.2	0	0.0	6	7.0	138	12.7	3	4.4	21	9.0	806	50.3	2	6.1	1	0.4	8	2.1	43	16.0	2	1.5	1125	16.0

**Table 3:** Characteristics of included patients according to RT-PCR result

	Influenza negative		Influenza positive			A (H1N1)pdm09			A (H3N2)			A not subtyped			B/Yamagata			B/Victoria			B not subtyped		
	*N* = 7245		*N* = 2895			*N* = 76			*N* = 1840			*N* = 129			*N* = 108			*N* = 618			*N* = 135		
Characteristic	n	%	n	%	P vs. negative	n	%	P vs. negative	n	%	P vs. negative	n	%	P vs. negative	n	%	P vs. negative	n	%	P vs. negative	n	%	P vs. negative
Age in years, median (range)	12 (0–105)		28 (0–103)		< 0.001	35 (0–84)		0.083	35 (0–103)		< 0.001	48 (0–102)		< 0.001	13 (0–92)		0.840	18 (0–89)		0.008	7 (0–94)		0.139
Age group					< 0.0001			0.0001			< 0.0001			< 0.0001			0.0003			< 0.0001			< 0.0001
0–1 y	2361	32.8	331	11.9		11	14.5		220	12.5		16	16.0		20	19.8		47	7.6		20	14.9	
2–4 y	906	12.6	311	11.2		12	15.8		162	9.2		13	13.0		16	15.8		86	13.9		24	17.9	
5–17 y	446	6.2	381	13.7		7	9.2		143	8.1		7	7.0		15	14.9		176	28.5		35	26.1	
18–49 y	1305	18.1	795	28.7		23	30.3		440	25.1		15	15.0		10	9.9		282	45.6		26	19.4	
50–64 y	540	7.5	195	7.0		13	17.1		159	9.1		3	3.0		12	11.9		5	0.8		4	3.0	
65–74 y	565	7.9	228	8.2		7	9.2		178	10.1		15	15.0		7	6.9		11	1.8		10	7.5	
75–84 y	631	8.8	272	9.8		3	3.9		223	12.7		16	16.0		11	10.9		9	1.5		12	9.0	
≥ 85 y	441	6.1	260	9.4		0	0.0		230	13.1		15	15.0		10	9.9		2	0.3		3	2.2	
Sex					< 0.0001			0.1374			< 0.0001			0.3877			0.6826			< 0.0001			0.5137
Male	3766	52.0	1339	46.3		33	43.4		859	46.7		72	55.8		54	50.0		254	41.1		74	54.8	
Female	3479	48.0	1556	53.7		43	56.6		981	53.3		57	44.2		54	50.0		364	58.9		61	45.2	
Chronic conditions					< 0.0001			0.1801			< 0.0001			< 0.0001			0.0025			< 0.0001			0.6485
0	4765	65.8	1661	57.4		44	57.9		894	48.6		51	39.5		58	53.7		528	85.4		92	68.1	
1	1240	17.1	572	19.8		19	25.0		415	22.6		27	20.9		18	16.7		71	11.5		24	17.8	
≥2	1240	17.1	662	22.9		13	17.1		531	28.9		51	39.5		32	29.6		19	3.1		19	14.1	
Previously hospitalised (last 12 months)					0.0163			0.2604			0.9969			0.6372			0.8445			0.0002			0.0086
No	5029	73.6	1659	76.2		58	79.5		924	73.6		44	71.0		53	72.6		494	80.5		94	84.7	
Yes	1802	26.4	518	23.8		15	20.5		331	26.4		18	29.0		20	27.4		120	19.5		17	15.3	
Underlying chronic conditions																							
Cardiovascular disease	1298	17.9	796	27.5	< 0.0001	17	22.4	0.3145	627	34.1	< 0.0001	60	46.5	< 0.0001	37	34.3	< 0.0001	30	4.9	< 0.0001	28	20.7	0.3970
Chronic obstructive pulmonary disease	802	11.1	206	7.1	< 0.0001	8	10.5	0.8806	159	8.6	0.0025	10	7.8	0.2328	10	9.3	0.5513	16	2.6	< 0.0001	7	5.2	0.0301
Asthma	276	3.8	187	6.5	< 0.0001	6	7.9	0.0656	147	8.0	< 0.0001	14	10.9	< 0.0001	8	7.4	0.0541	8	1.3	0.0013	4	3.0	0.6100
Immunodeficiency/organ transplant	155	2.1	67	2.3	0.5867	3	3.9	0.2806	49	2.7	0.1758	7	5.4	0.0116	3	2.8	0.6497	2	0.3	0.0020	3	2.2	0.9475
Diabetes	687	9.5	362	12.5	< 0.0001	11	14.5	0.1405	292	15.9	< 0.0001	33	25.6	< 0.0001	13	12.0	0.3693	5	0.8	< 0.0001	8	5.9	0.1610
Renal impairment	409	5.6	208	7.2	0.0034	4	5.3	0.8858	161	8.8	< 0.0001	11	8.5	0.1616	7	6.5	0.7089	19	3.1	0.0069	7	5.2	0.8184
Neuromuscular disease	234	3.2	192	6.6	< 0.0001	2	2.6	0.7690	147	8.0	< 0.0001	15	11.6	< 0.0001	8	7.4	0.0157	12	1.9	0.0775	9	6.7	0.0266
Neoplasm	311	4.3	168	5.8	0.0012	0	0.0	0.0649	133	7.2	< 0.0001	20	15.5	< 0.0001	7	6.5	0.2670	4	0.6	< 0.0001	5	3.7	0.7377
Cirrhosis/liver disease	97	1.3	38	1.3	0.9171	0	0.0	0.3099	29	1.6	0.4372	3	2.3	0.3369	1	0.9	0.7103	4	0.6	0.1428	1	0.7	0.5475
Autoimmune disease	96	1.3	35	1.2	0.6402	1	1.3	0.9944	16	0.9	0.1139	1	0.8	0.5869	4	3.7	0.0341	12	1.9	0.2061	1	0.7	0.5548
Pregnant (women 15–45 y)	459	58.0	481	82.7	< 0.0001	1	10.0	0.0023	272	83.7	< 0.0001	2	28.6	0.1164	1	14.3	0.0198	196	89.9	< 0.0001	9	56.3	0.8866
Obese (all ages)	1083	15.6	374	14.6	0.1967	18	25.4	0.0250	271	17.0	0.1905	13	17.1	0.7231	17	18.3	0.4834	46	7.4	< 0.0001	12	9.6	0.0654
Outpatient consultations last 3 months					0.6362			0.7448			0.0005			0.7360			0.0061			0.0120			0.0008
0	2504	36.7	779	35.8		25	34.2		388	30.9		20	32.3		40	54.8		262	42.7		48	43.2	
1	1448	21.2	480	22.0		14	19.2		287	22.9		15	24.2		11	15.1		121	19.7		35	31.5	
≥ 2	2879	42.1	918	42.2		34	46.6		580	46.2		27	43.5		22	30.1		231	37.6		28	25.2	
Smoking habits (patients ≥18 y)					< 0.0001			0.0753			< 0.0001			0.1387			0.9041			0.1663			0.0818
Never smoker	4106	57.0	1598	57.5		42	56.0		993	56.7		62	53.9		57	56.4		367	59.5		84	64.1	
Past smoker	1366	19.0	640	23.0		21	28.0		459	26.2		23	20.0		18	17.8		98	15.9		15	11.5	
Current smoker	1728	24.0	542	19.5		12	16.0		300	17.1		30	26.1		26	25.7		152	24.6		32	24.4	
Functional status impairment (Barthel score; patients ≥65 y)					0.0764			0.5686			0.1750			0.9911			0.4228			0.6788			0.0012
Total (0–15)	106	6.8	24	3.9		0	0.0		21	4.2		3	7.3		0	0.0		0	0.0		0	0.0	
Severe (20–35)	35	2.3	15	2.4		0	0.0		11	2.2		1	2.4		0	0.0		0	0.0		3	13.0	
Moderate (40–55)	62	4.0	31	5.0		0	0.0		26	5.2		1	2.4		0	0.0		1	4.5		3	13.0	
Mild (60–90)	364	23.5	136	22.1		4	44.4		109	21.9		10	24.4		6	24.0		5	22.7		3	13.0	
Minimal (95–100)	985	63.5	409	66.5		5	55.6		330	66.4		26	63.4		19	76.0		16	72.7		14	60.9	
Sampling time					< 0.0001			0.0051			< 0.0001			0.0797			0.7704			< 0.0001			0.3919
0–2 days	2374	33.1	1211	42.0		16	21.1		830	45.3		54	41.9		35	32.7		237	38.3		40	29.6	
3–4 days	2521	35.2	1052	36.5		22	28.9		657	35.9		38	29.5		41	38.3		244	39.5		53	39.3	
5–7 days	1941	27.1	564	19.5		34	44.7		303	16.5		35	27.1		28	26.2		132	21.4		39	28.9	
8–9 days	335	4.7	59	2.0		4	5.3		42	2.3		2	1.6		3	2.8		5	0.8		3	2.2	
Influenza vaccination ≥15 days from symptom onset	938	13.0	279	9.6	< 0.0001	7	9.2	0.3339	221	12.0	0.2825	10	7.8	0.0806	9	8.3	0.1554	25	4.1	< 0.0001	8	5.9	0.0156
Influenza vaccination ≥15 days from symptom onset (age ≥ 65)	673	39.9	195	22.1	< 0.0001	1	10.0	0.0541	175	24.4	< 0.0001	8	10.7	< 0.0001	6	17.1	0.0064	1	4.6	0.0008	4	15.4	0.0112
Influenza vaccination ≥15 days from symptom onset (targeted groups)	869	18.4	256	11.1	< 0.0001	7	13.0	0.3047	214	13.6	< 0.0001	8	7.2	0.0025	7	11.1	0.1373	14	3.1	< 0.0001	7	9.7	0.0586

Patients with a qualified occupation had a higher risk of being admitted with influenza. Patients with a swab taken 8–9 days after symptoms onset appeared with less risk of being admitted with influenza, suggesting a decrease in the influenza viral load for these patients (Table 4).

**Table 4:** Subject characteristics and risk of admission with influenza

	All admissions	Influenza-positive		Crude OR		Heterogeneity by strain (I^2^)	aOR^(*)^	
	N = 10140	N = 2895						
Characteristic	N	N	%	Value	95% CI		Value	95% CI
Age group								
0–1 years	2692	331	12.3	1.00	–	79.4%	1.00	–
2–4 years	1217	311	25.6	2.45	2.06–2.92	75.6%	0.86	0.67–1.09
5–17 years	827	381	46.1	6.09	5.03–7.38	94.6%	1.59	0.85–2.96
18–49 years	2100	795	37.9	4.35	3.73–5.06	96.4%	0.65	0.22–1.97
50–64 years	735	195	26.5	2.58	2.10–3.15	96.6%	0.59	0.25–1.39
65–74 years	793	228	28.8	2.88	2.37–3.50	95.3%	0.61	0.31–1.22
75–84 years	903	272	30.1	3.07	2.55–3.71	96.9%	0.50	0.21–1.20
≥ 85 years	701	260	37.1	4.21	3.45–5.13	98.4%	0.49	0.19–1.28
Sex								
Male	5105	1339	26,2%	1.00		54.0%	1.00	
Female	5035	1556	30,9%	1.26	1.15–1.37	46.5%	0.84	0.74–0.95
Smoking habits								
Current smoker	2270	542	23,9%	1.00		81.7%	1.00	
Past smoker	2006	640	31,9%	1.49	1.30–1.71	88.4%	1.04	0.89–1.22
Never smoker	5704	1598	28,0%	1.24	1.11–1.39	34.0%	1.09	0.93–1.28
Consultations at the GP (last 3 months)								
No	3283	779	23,7%	1.00		95.0%	1.00	
Yes	5725	1398	24,4%	1.04	0.94–1.15	92.6%	0.91	0.69–1.18
Occupation / Social class								
Qualified	3810	1255	32,9%	1.00		97.1%	1.00	
Skilled	1376	355	25,8%	0.71	0.62–0.81	81.9%	0.83	0.72–0.94
Low or unskilled	3411	591	17,3%	0.43	0.38–0.48	91.5%	0.63	0.50–0.78
Other risk factors								
Comorbidity	3714	1234	33,2%	1.43	1.31–1.56	98.7%	0.90	0.63–1.30
Cardiovascular disease	2094	796	38,0%	1.74	1.57–1.92	98.7%	1.01	0.72–1.40
Chronic obstructive pulmonary disease	1008	206	20,4%	0.62	0.52–0.72	92.5%	0.66	0.45–0.98
Asthma	463	187	40,4%	1.74	1.44–2.11	94.3%	1.31	0.96–1.77
Immunodeficiency/organ transplant	222	67	30,2%	1.08	0.81–1.45	85.2%	0.57	0.28–1.17
Diabetes	1049	362	34,5%	1.36	1.19–1.56	98.1%	1.19	1.03–1.37
Chronic renal impairment	617	208	33,7%	1.29	1.09–1.54	89.2%	1.06	0.89–1.27
Chronic neuromuscular disease	426	192	45,1%	2.13	1.75–2.59	91.7%	1.08	0.75–1.56
Active neoplasm	479	168	35,1%	1.37	1.13–1.67	96.8%	0.63	0.42–0.95
Chronic liver disease	135	38	28,1%	0.98	0.67–1.43	38.8%	1.09	0.79–1.50
Autoimmune disease	131	35	26,7%	0.91	0.62–1.35	23.8%	1.14	0.84–1.56
Obesity	1457	374	25,7%	0.92	0.81–1.04	93.3%	0.83	0.69–1.00
Pregnancy	942	483	51,3%	2.96	2.58–3.40	97.6%	3.02	1.59–5.76
Days from onset of symptoms to swabbing								
0–2 days	3585	1211	33,8%	1.00		92.8%	1.00	
3–4 days	3573	1052	29,4%	0.82	0.74–0.90	36.9%	1.05	0.99–1.12
5–7 days	2505	564	22,5%	0.57	0.51–0.64	83.4%	0.82	0.64–1.07
8–9 days	394	59	15,0%	0.35	0.26–0.46	65.2%	0.60	0.47–0.77

^(*)^Adjusted Odds Ratios were obtained using the model described in the ‘Methods’ section (pg.6)

**Fig. 6:**
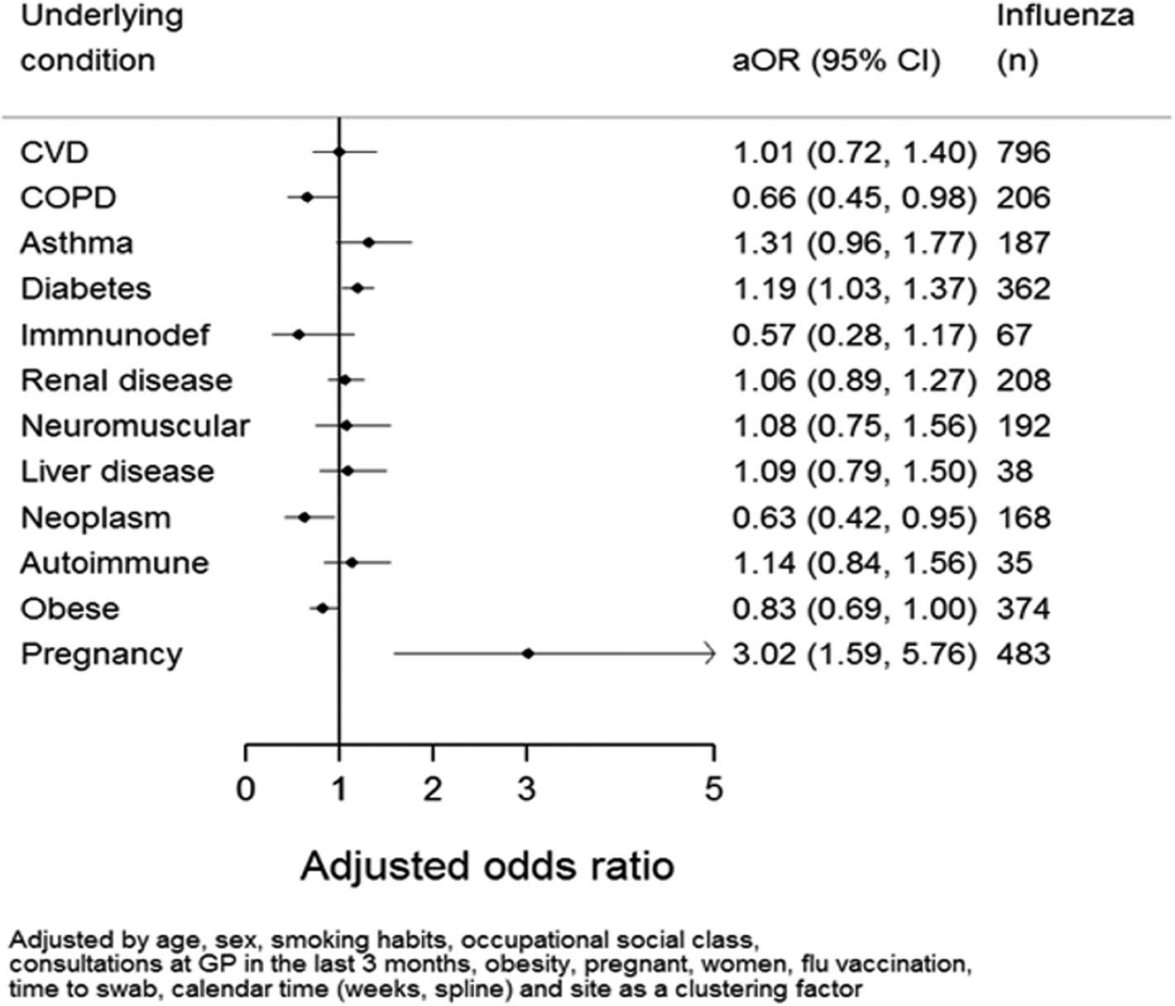
Adjusted Odds Ratio (aOR) and number of admissions with influenza according to comorbidity

Pregnant women had a 3 times higher risk of having influenza at admission than non-pregnant. Also subjects with diabetes had 1.19 times higher risk of being an influenza case. On the other hand, patients with COPD or neoplasm had lower risk of testing positive for influenza. Despite there was a high number of admissions with cardiovascular diseases (CVD), no difference in the risk of influenza was found in these patients. (Fig. 6).

**Fig. 7:**
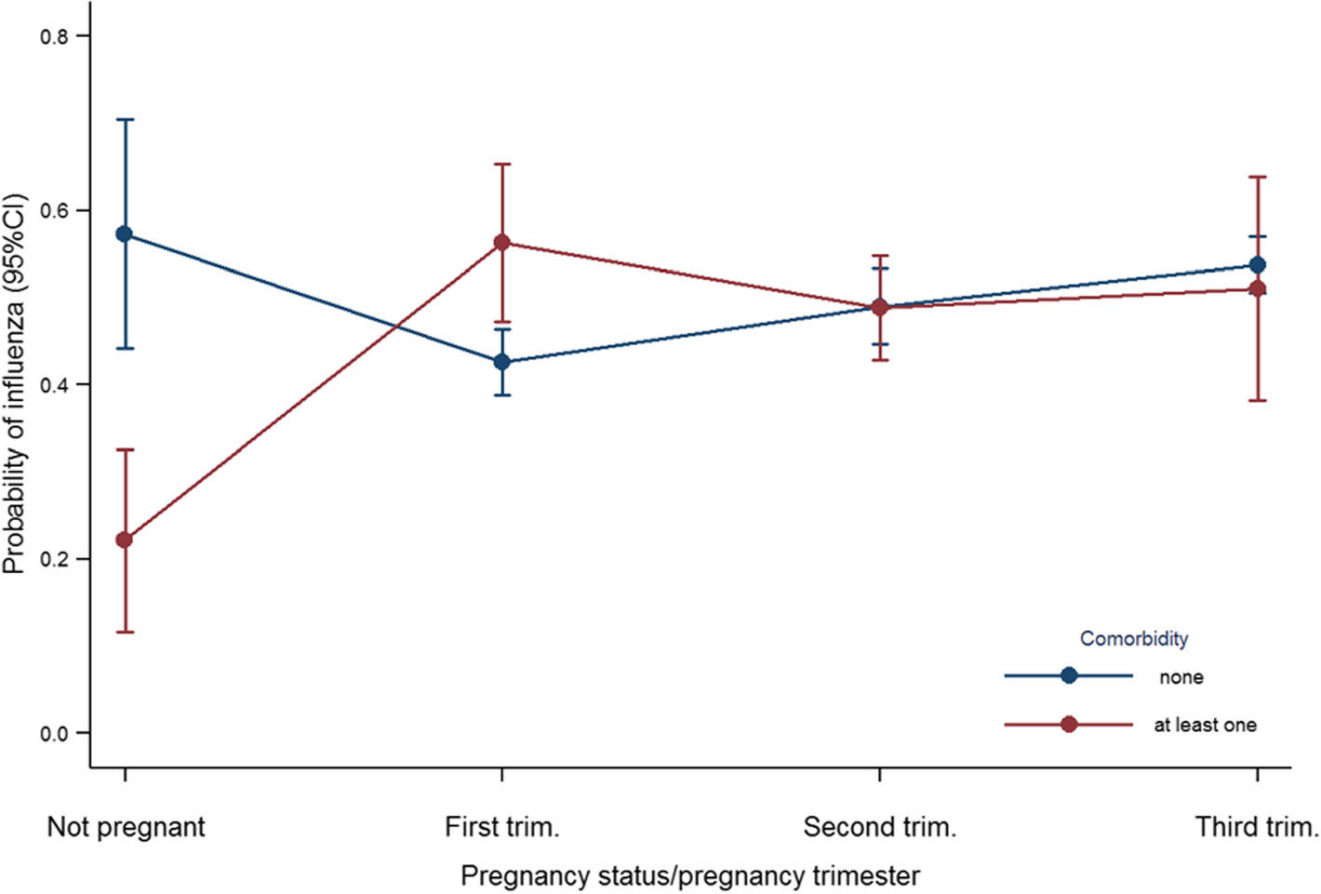
Predicted probability of having an admission with influenza in pregnant and non-pregnant women by trimester

During pregnancy, the risk of testing positive for influenza was higher during the third trimester than in the first trimester, and also if they had any comorbidity in the first trimester (Fig. 7).

There were no significant statistical differences among influenza positives and negatives for those who were admitted to ICU or who received mechanical ventilation or those who died while they were hospitalised, and differences for those with extracorporeal membrane oxygenation could be due to sparse numbers of patients who received extracorporeal membrane oxygenation. Apart from influenza, the main discharge diagnosis was pneumonia, either for influenza-negatives or influenza-positives (Table 5).

**Table 5:** Influenza severity and complications 232 by RT-PCR results

	Influenza-negative		Influenza-positive			A(H1N1)pdm09		A (H3N2)		A not subtyped		B/Yamagata		B/Victoria		B not subtyped		
	N=7245		N=2895			N=76		N=1840		N=129		N=108		N=618		N=135		
Category	n	%	n	%	P vs. negative	n	%	n	%	n	%	n	%	n	%	n	%	P-value for distribution by strain
Severity indicator																		
Intensive care unit admission	317	4.4	132	4.6	0.6656	9	11.8	102	5.5	5	3.9	5	4.6	6	1.0	6	4.4	<0.0001
Mechanical ventilation	225	3.1	75	2.6	0.1728	5	6.6	61	3.3	3	2.3	2	1.9	3	0.5	2	1.5	0.0018
Extracorporeal membrane oxygenation	89	1.2	9	0.3	0.0000	0	0.0	5	0.3	3	2.3	0	0.0	1	0.2	0	0.0	0.0035
Death during hospitalisation	183	2.5	69	2.4	0.6904	4	5.3	52	2.8	3	2.3	3	2.8	5	0.8	2	1.5	0.0745
Length of stay (days), median (interquartile range)	6	(3-8)	5	(3-8)	<0.001	6	(3-10)	5	(3-8)	6	(3-9)	4	(2-6.5)	6	(4-8)	5	(3-7)	0.004
Respiratory diagnoses					<0.0001													0.3163
None	2052	28.3	1828	63.1		15	19.7	1191	64.7	79	61.2	51	47.2	435	70.4	60	44.4	
Pneumonia	2335	32.2	658	22.7		58	76.3	362	19.7	37	28.7	40	37.0	112	18.1	55	40.7	
COPD exacerbation	192	2.7	91	3.1		2	2.6	74	4.0	5	3.9	3	2.8	3	0.5	4	3.0	
Respiratory failure	109	1.5	12	0.4		1	1.3	9	0.5	1	0.8	0	0.0	0	0.0	1	0.7	
Asthma exacerbation	53	0.7	30	1.0		0	0.0	29	1.6	0	0.0	0	0.0	1	0.2	0	0.0	
Acute respiratory distress syndrome	18	0.2	2	0.1		0	0.0	0	0.0	0	0.0	0	0.0	2	0.3	0	0.0	
Pneumotorax	1	0.0	0	0.0		0	0.0	0	0.0	0	0.0	0	0.0	0	0.0	0	0.0	
Bronchiolitis	383	5.3	48	1.7		0	0.0	29	1.6	1	0.8	0	0.0	12	1.9	6	4.4	
Upper respiratory infection	2101	29.0	226	7.8		0	0.0	146	7.9	6	4.7	14	13.0	53	8.6	9	6.7	
Metabolic failure					0.1725													0.2106
No	7016	96.8	2827	97.7		72	94.7	1803	98.0	126	97.7	106	98.1	604	97.7	127	94.1	
Acute renal failure	85	1.2	19	0.7		3	3.9	10	0.5	2	1.6	2	1.9	0	0.0	2	1.5	
Diabetic coma	8	0.1	1	0.0		0	0.0	1	0.1	0	0.0	0	0.0	0	0.0	0	0.0	
Fluid/electrolyte/acid-base/balance disorders	136	1.9	48	1.7		1	1.3	26	1.4	1	0.8	0	0.0	14	2.3	6	4.4	
Cardiovascular events					<0.0001													<0.0001
None	6674	92.1	2766	95.5		69	90.8	1741	94.6	122	94.6	104	96.3	611	98.9	129	95.6	
Acute myocardial infarction	6	0.1	1	0.0		0	0.0	1	0.1	0	0.0	0	0.0	0	0.0	0	0.0	
Arterial or venous embolia	1	0.0	0	0.0		0	0.0	0	0.0	0	0.0	0	0.0	0	0.0	0	0.0	
Carditis	2	0.0	1	0.0		0	0.0	0	0.0	0	0.0	0	0.0	1	0.2	0	0.0	
Cardiac arrest	1	0.0	1	0.0		0	0.0	1	0.1	0	0.0	0	0.0	0	0.0	0	0.0	
Malignant hypertension	1	0.0	3	0.1		0	0.0	2	0.1	0	0.0	0	0.0	0	0.0	1	0.7	
Any cardiovascular condition	560	7.7	123	4.2		7	9.2	95	5.2	7	5.4	4	3.7	6	1.0	5	3.7	
Neurologic events					0.4268													0.4345
No	7241	99.9	2894	100.0		76	100.0	1839	99.9	129	100.0	108	100.0	618	100.0	135	100.0	
Altered mental status	3	0.0	1	0.0		0	0.0	1	0.1	0	0.0	0	0.0	0	0.0	0	0.0	
Convulsions	1	0.0	0	0.0		0	0.0	0	0.0	0	0.0	0	0.0	0	0.0	0	0.0	
Major discharge diagnoses					<0.0001													<0.0001
Influenza	241	3.3	2272	78.5		40	52.6	1401	76.1	97	75.2	39	36.1	584	94.5	113	83.7	
Pneumonia	2427	33.5	238	8.2		31	40.8	145	7.9	12	9.3	29	26.9	13	2.1	12	8.9	
Other respiratory disease	2683	37.0	177	6.1		1	1.3	132	7.2	8	6.2	15	13.9	17	2.8	6	4.4	
Cardiovascular	267	3.7	34	1.2		1	1.3	31	1.7	1	0.8	1	0.9	0	0.0	1	0.7	
Other	1627	22.5	174	6.0		3	3.9	131	7.1	11	8.5	24	22.2	4	0.6	3	2.2	

**Fig. 8:**
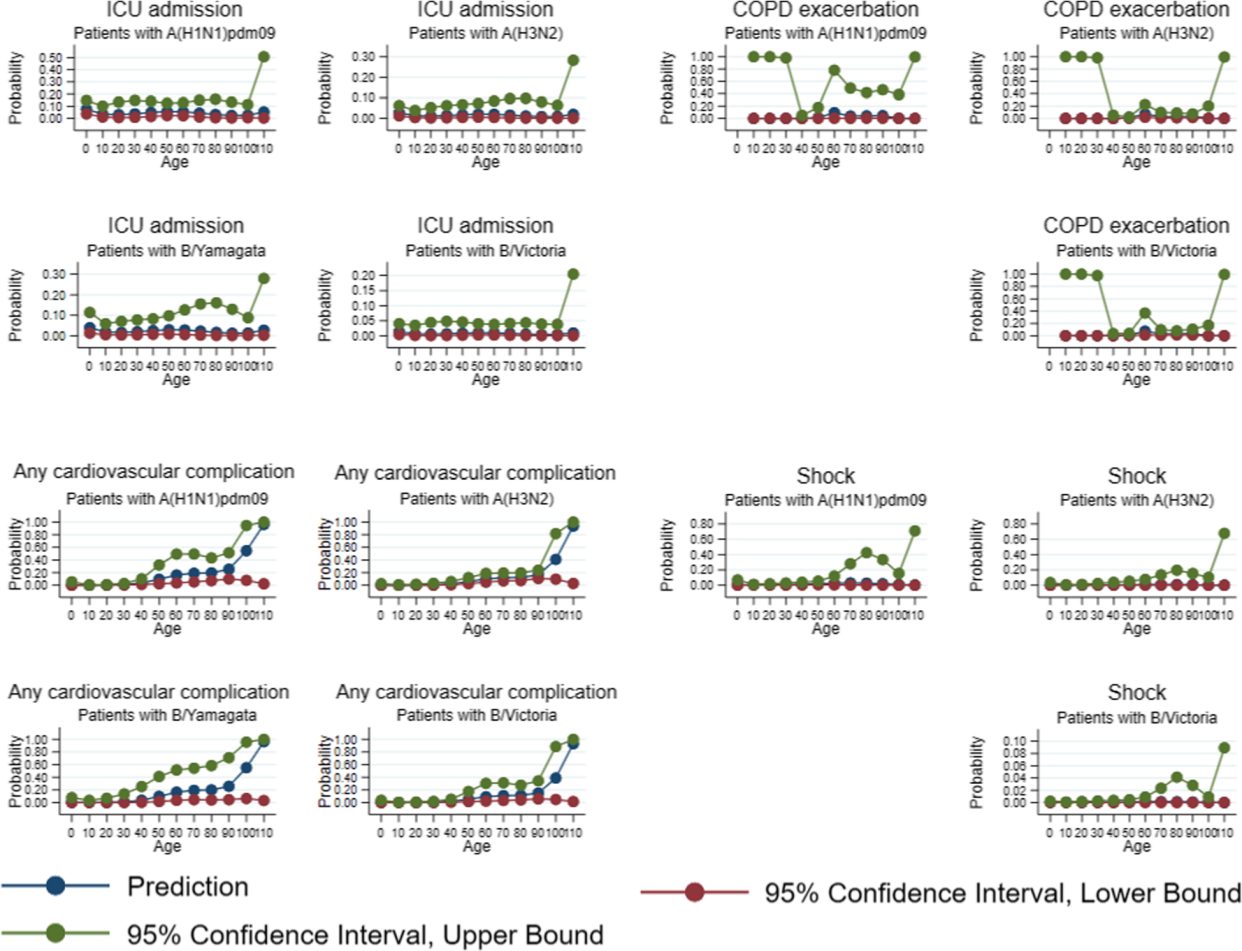
Predicted probability of severe outcome by strain

Probabilities of most common severe outcomes by strain by age and influenza strains are displayed in Fig. 8. This probability had an upward trend up to 80 years old after a shock. The probability point estimates of having any cardiovascular complication increased greatly from 90 years old for those who had influenza. Similar trends were found for each individual strain for these discharge diagnoses.

**Fig. 9:**
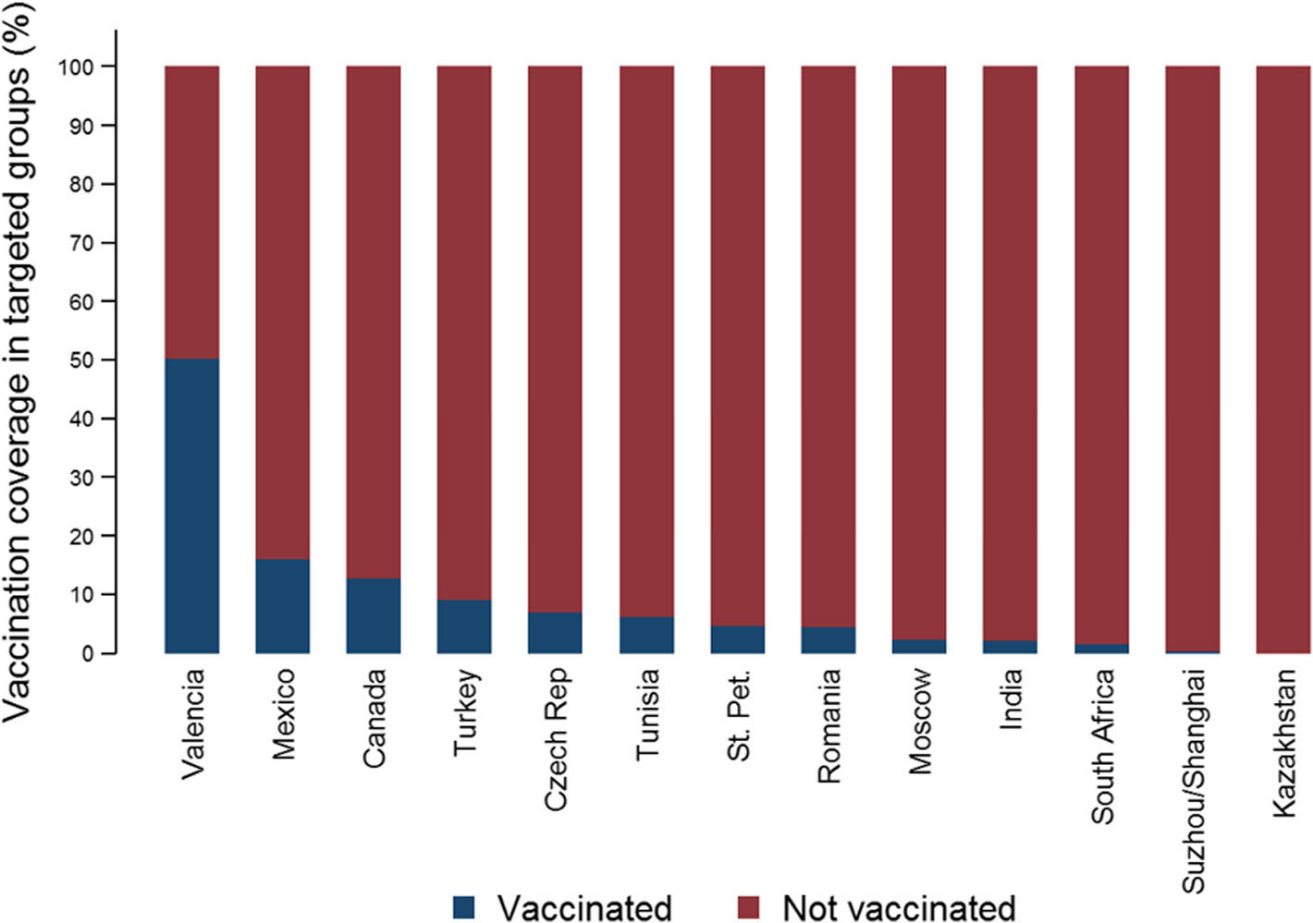
Vaccination coverage in targeted groups by site

Vaccination coverage was 9% or higher for targeted groups only in 4 sites (Fig. 9), and only 6 sites had at least 20 patients vaccinated among the patients targeted for vaccination. The IVE analysis was restricted to the sites with the highest vaccination coverage in targeted groups for vaccination having at least 20 patients vaccinated in these groups. These sites were Valencia, Canada, St. Petersburg, Mexico, Moscow and Turkey.

The IVE analysis, therefore, will be carried out in these six sites and globally. Vaccination coverage in pregnant women was 0% in Kazakhstan among the included patients, and in Moscow, only 1.3% (10 out of 800) of the admitted pregnant women received the vaccine at least 15 days before symptoms onset, therefore, adjusted IVE could not be estimated for pregnant women.

Vaccination coverage was higher in patients older than 65 years and in patients with two or more comorbidities. Among immunized women 15 to 45 years old, 19 of 47 were pregnant (40.4%), and among all vaccinated patients, 26.7% were obese.

Of the subjects vaccinated, 78.0% were also vaccinated in season 2015–2016 and 67.2% were vaccinated in season 2014–2015. However, 8.0% of the unvaccinated patients in the current season were vaccinated in the season 2015–2016, and 6.6% in the season 2014–2015 (Table 6).

**Table 6:** Characteristics of patients included in the primary analysis by vaccination status

Risk variables		Unvaccinated		Vaccinated		*P* value
	Category	n	%	n	%	
Number of patients, n (%)	Controls	6307	70.7	938	77.1	< 0.0001
	Cases	2616	29.3	279	22.9	
Age (y)	Median (range)	11.4 (0–105.3)		76.5 (0.6–102.8)		< 0.0001
Age group, n (%) ^(2)^	0–5 months	1254	14.3%	0	0.0%	< 0.0001
	6–11 months	643	7.3%	13	1.1%	
	1–4 yrs	1948	22.2%	51	4.3%	
	5–17 yrs	760	8.7%	67	5.6%	
	18–49 yrs	1988	22.7%	112	9.4%	
	50–64 yrs	628	7.2%	106	8.9%	
	65–74 yrs	583	6.6%	210	17.6%	
	75–84 yrs	566	6.5%	337	28.2%	
	≥85 y	403	4.6%	299	25.0%	
Sex, n (%)	Male	4462	50.0%	643	52.8%	0.0641
	Female	4461	50.0%	574	47.2%	
Comorbidities, n (%)	None	6123	68.6%	303	24.9%	< 0.0001
	1	1457	16.3%	355	29.2%	
	> 1	1343	15.1%	559	45.9%	
Pregnant, n (%)	–	921	69.5%	19	40.4%	< 0.0001
Obesity, n (%)	–	1148	13.8%	309	26.7%	< 0.0001
Previous hospitalisation within 12 months, n (%)	–	1914	24.1%	406	37.7%	< 0.0001
GP visit within 3 months, n (%)	None	3074	38.8%	209	19.4%	< 0.0001
	1	1740	21.9%	188	17.4%	
	> 1	3116	39.3%	681	63.2%	
Smoking, n (%)	Current	2112	24.1%	158	13.0%	< 0.0001
	Past	1618	18.5%	388	32.0%	
	Never	5037	57.5%	667	55.0%	
Functional impairment in ≥65 y, n (%)	None or minimal	72	5.4%	58	7.0%	0.4086
	Mild	32	2.4%	18	2.2%	
	Moderate	52	3.9%	41	4.9%	
	Severe	309	23.1%	191	23.0%	
	Total	871	65.2%	523	62.9%	
Sampling interval (days)	Median (range)	3 (0–9)		4 (0–9)		< 0.0001
Sampling interval, n (%)	≤4 days	6377	72.1%	781	64.2%	< 0.0001
	5–7 days	2148	24.3%	357	29.3%	
	8–9 days	315	3.6%	79	6.5%	
Site, n (%)	St. Pet	1851	20.7%	86	7.1%	< 0.0001
	Moscow	1555	17.4%	65	5.3%	
	Kazakhstan	159	1.8%	0	0.0%	
	Czech Republic	105	1.2%	6	0.5%	
	Canada	993	11.1%	139	11.4%	
	Romania	380	4.3%	7	0.6%	
	Turkey	392	4.4%	21	1.7%	
	Valencia	1300	14.6%	825	67.8%	
	Tunisia	37	0.4%	2	0.2%	
	Suzhou/Shanghai	469	5.3%	1	0.1%	
	India	482	5.4%	11	0.9%	
	Mexico	301	3.4%	49	4.0%	
	South Africa	899	10.1%	5	0.4%	
Vaccinated, n (%)	In 2015–2016	718	8.0%	949	78.0%	< 0.0001
	In 2014–2015	589	6.6%	818	67.2%	< 0.0001

### IVE estimates for included patients

**Fig. 10:**
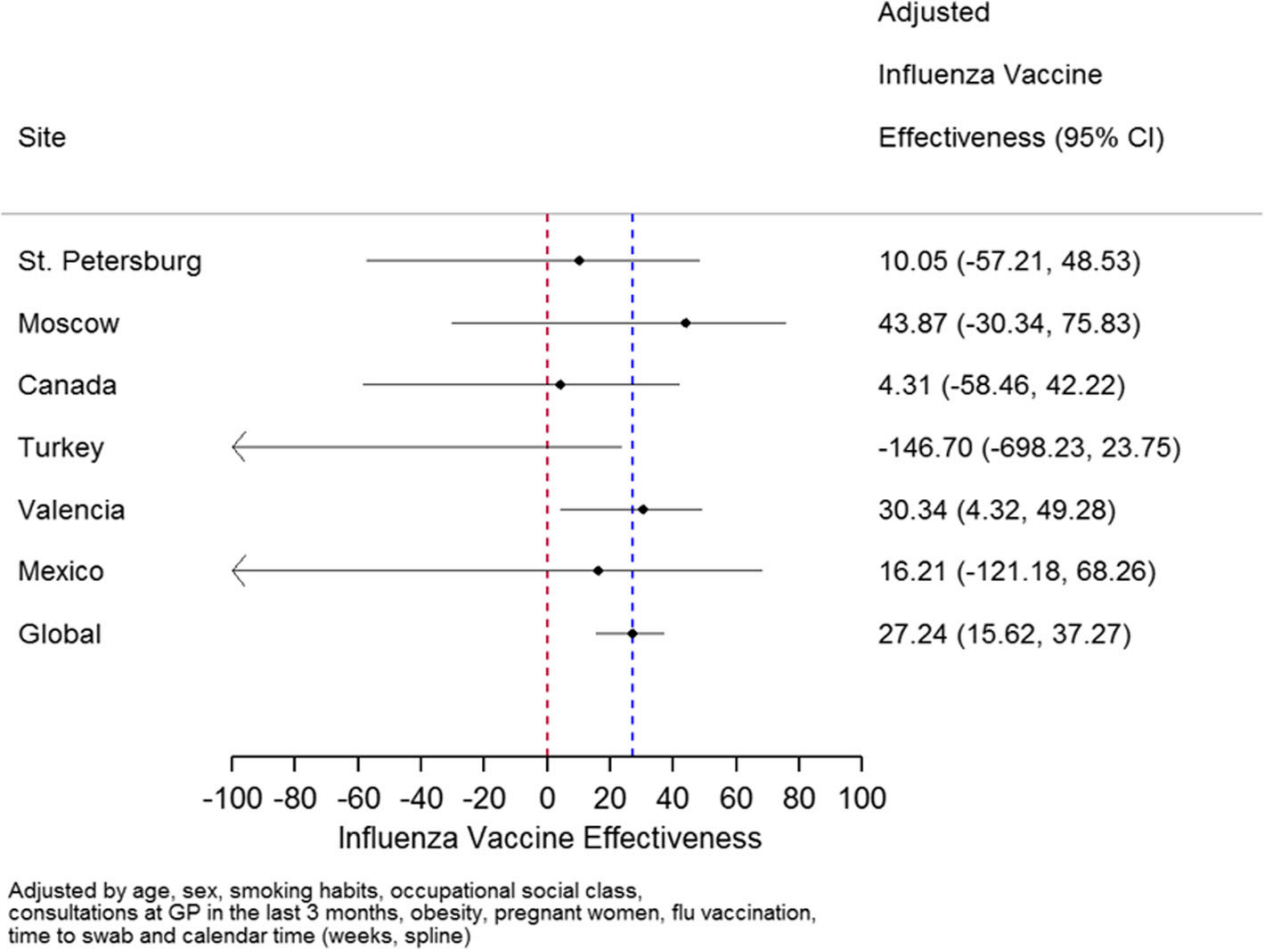
Adjusted Influenza Vaccine Effectiveness by site

In the selected sites for IVE estimates, vaccination coverage was 11.7% among the influenza positives and 22.2% among the influenza negatives. The overall IVE was 27.24% (95% CI 15.62 to 37.27%) in targeted groups for vaccination. Table 7 shows IVE for different strains, Fig. 10 by study country.

**Table 7:** IVE for all cases and for targeted groups only by age and strain

			Influenza-positive		Influenza-negative		Adjusted IVE^(*)^	
Population	Strain	Age	Total	Vaccinated	Total	Vaccinated	Percent (95% CI)	P-value
Overall	Any	Any	2895	279	7245	938	27 (15, 38)	
		<65 y	2013	84	5558	265	27 (−1, 48)	0.804
		≥65 y	882	195	1687	673	25 (3, 43)	
	A (H1N1) pdm09	Any	76	7	7245	938	39 (−68, 78)	
		<65 y	66	6	5558	265	2 (−138, 60)	0.346
		≥65 y	10	1	1687	673	99 (1, 100)	
	A (H3N2)	Any	1840	221	7245	938	25 (13, 35)	
		<65 y	1124	46	5558	265	31 (1, 51)	0.703
		≥65 y	716	175	1687	673	19 (−10, 40)	
	B/Yamagata	Any	108	9	7245	938	41 (−110, 84)	
		<65 y	73	3	5558	265	7 (−178, 69)	0.203
		≥65 y	35	6	1687	673	73 (−38, 95)	
	B/Victoria	Any	618	25	7245	938	43 (−15, 71)	
		<65 y	596	24	5558	265	27 (−14, 54)	0.191
		≥65 y	22	1	1687	673	89 (40, 98)	
Targeted groups only	Any	Any	2314	256	4723	869	27 (16, 37)	
		<65 y	1432	61	3036	196	37 (0, 47)	0.657
		≥65 y	882	195	1687	673	25 (3, 43)	
	A (H1N1) pdm09	Any	54	7	4723	869	18 (−142, 72)	
		<65 y	44	6	3036	196	−62 (−303, 35)	0.423
		≥65 y	10	1	1687	673	99 (1, 100)	
	A (H3N2)	Any	1572	214	4723	869	23 (9, 34)	
		<65 y	856	39	3036	196	27 (−7, 50)	0.485
		≥65 y	716	175	1687	673	19 (−10, 40)	
	B/Yamagata	Any	63	7	4723	869	72 (8, 92)	
		<65 y	28	1	3036	196	65 (−35, 91)	0.037
		≥65 y	35	6	1687	673	73 (−38, 95)	
	B/Victoria	Any	449	14	4723	869	66 (3, 80)	
		<65 y	427	13	3036	196	41 (10, 62)	0.262
		≥65 y	22	1	1687	673	89 (40, 98)	

^(*) .^IVE was obtained in each case using the same model (described in the ‘Methods’ section) but restricting it by strain, age or targeted groups.. P-value obtained comparing patients <65 y and ≥ 65 y

**Fig. 11:**
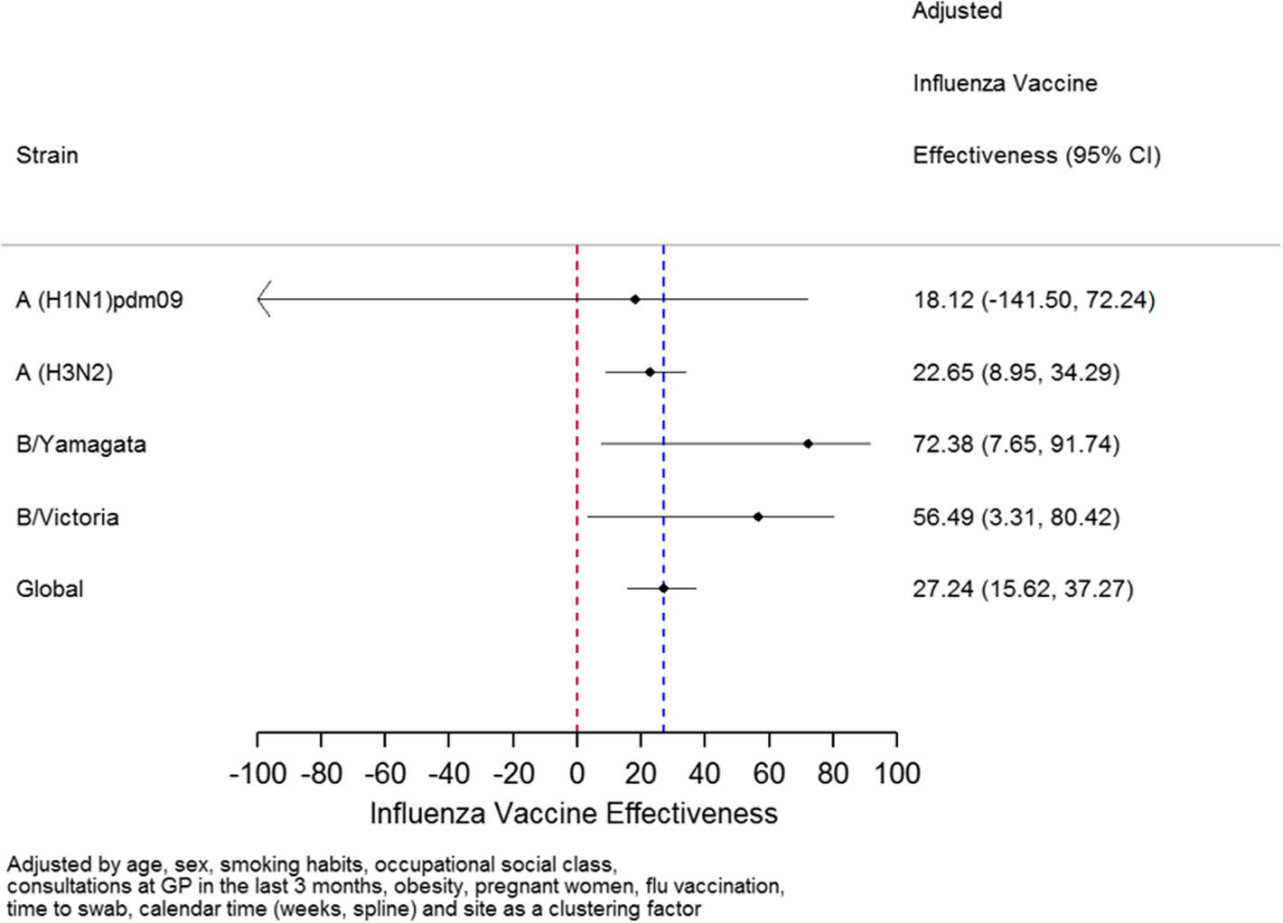
Adjusted Influenza Vaccine Effectiveness by strain

IVE was statistically significant for all strains except for A(H1N1)pdm09 due to the limited sample size, and the point estimate was higher for both influenza B lineages, even using the trivalent vaccine (Fig. 11). Heterogeneity among influenza types/subtypes was relevant (I^2^ = 57.4%).

**Fig. 12:**
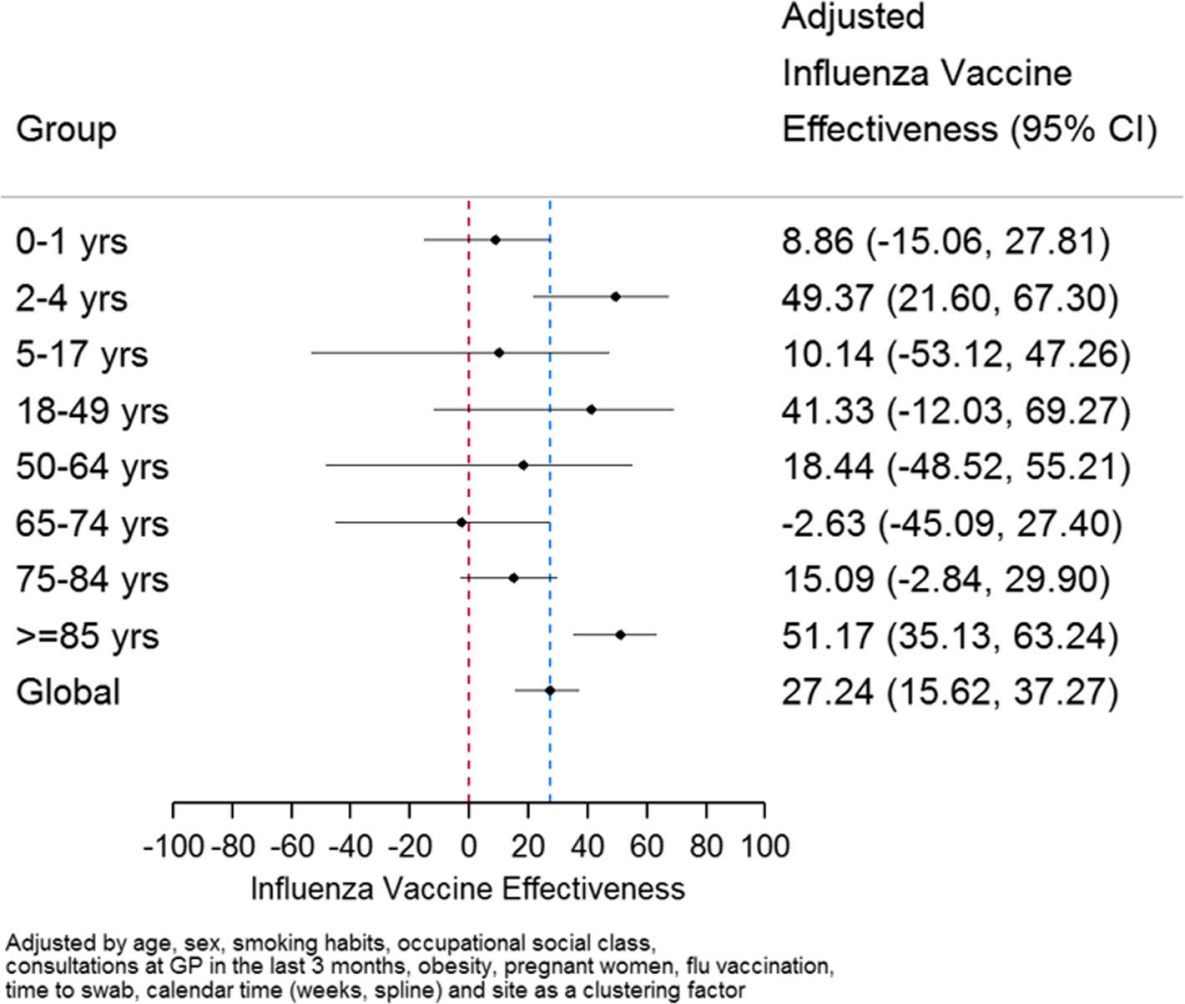
Adjusted Influenza Vaccine Effectiveness by age group

This season IVE estimate was higher in patients 85 years old or older (51.17% [95% CI: 35.13 to 63.24]). IVE was also high and statistically significant for patients 2 to 4 years old (49.37% [95% CI: 21.60 to 67.30]) (Fig. 12). Heterogeneity among the different age groups was relevant (I^2^ = 69%).

## Discussion

The GIHSN included sites from the two hemispheres in the 2016/17 season. However, Ivory Coast and Peru were not included in the epidemiology study or in the IVE study due to the low influenza cases detected. This season was characterized by a predominance in the circulation of A(H3N2) virus, and a second wave of B/Victoria. However, A(H1N1)pdm09 was predominant in Mexico. B/Yamagata-strain, which was not included in the vaccine, also circulated in some areas.

Influenza A(H1N1)pdm09 was mainly found in Mexico. A low vaccination coverage was seen in most of the GIHSN sites.

The GIHSN represents an opportunity to analyse the epidemiology of hospitalized influenza cases, and an assessment of the vaccine effectiveness worldwide. However, there are some limitations that should be mentioned:

- Although the same protocol was developed, the adaptation to different countries or sites produced some heterogeneity in the results, as previously reported in the network [3].
- In general vaccination coverage was low in most sites, even among high risk groups.
- Other factors as number of cases per site, and variability in the vaccination coverage, increased the heterogeneity in the reporting and analysis.

All of these limitations contributed to the complexity of the interpretation of the results.

In the northern hemisphere, the season differed by latitude [14], and this may have implications in the calendar of the vaccination campaigns.

Patients tested for influenza 8 to 9 days after symptoms onset had a higher proportion of samples negative for influenza than patients tested within the first 7 days after symptoms onset, as that viral load decreases with increasing time since infection, [15]. However, there were a few cases in our study as we collected all cases whose admission was in the 7 days after ILI symptoms started, and any delay in approaching the patient could result in a late swabbing.

Among inpatients with COPD, there was not a higher risk of testing for influenza. As all the cases were hospitalized, this result cannot be interpreted as COPD not being a risk factor for influenza hospitalization, as any other respiratory infection may decompensate the respiratory condition and force an admission. Besides vaccination coverage is higher in subjects with chronic conditions [16] and therefore, protection from the vaccine may also impact on our finding.

The risk of testing positive for influenza in diabetic patients was slightly higher than non-diabetic patients, as it also happened in previous seasons [3, 4]. Pregnancy also increased the probability of having influenza in women, particularly if they had at least one comorbidity in the first trimester.

Despite differences in the characteristics of the included patients relative to the age or pregnancy status, heterogeneity in the IVE analysis among the 6 sites with the highest numbers of vaccinated patients was low. Point estimates of the overall IVE from a two-step pooling was 27.2% (95% CI: 15.62 to 37.27) in hospitalized, which is higher than that reported in Europe for hospitalised patients [17], that ranged from 2.4 to 7.9%, depending on the age group, and lower to that estimated by the US CDC, which was 40% (95% CI: 32 to 46) [18].

Pooled Influenza vaccine effectiveness showed protection against all influenza virus that circulated, although for A(H1N1)pdm09 did not reach statistical significance, as the circulation of the virus was low except in Mexico. There was a significant effectiveness against both B lineages, even though most of the vaccines used were trivalent, i.e. only contained the B/Victoria linage, following recommendations of the World Health Organisation (WHO) for trivalent vaccines in the Northern Hemisphere [19]. Although antigenically different, there has been shown some degree of cross-protection among both B lineages.

## Conclusion

The GIHSN provides an opportunity to analyse influenza epidemiology and vaccine effectiveness worldwide. In the 2016/17 season, A(H3N2) was the predominant influenza strain this season (first wave), followed by B/Victoria (second wave). Influenza A(H1N1)pdm09 was mainly found in Mexico. A low vaccination coverage was seen in most of the GIHSN sites.

Differences in the distribution of influenza cases among the age groups were mainly due to the characteristics of the participating hospitals. Pregnant women had higher risk of testing positive for influenza, as occurred with diabetics, however this difference was not seen in COPD subjects.

Overall IVE was low to moderate 27.24 (95% CI 15.62 to 37.27) in this season. A moderate to high effectiveness was seen for both influenza B lineages, and a non-significant low effectiveness for Influenza A(H1N1)pdm09.

## Additional file

Additional file 1: Complementary **Table S1**. (DOCX 142 kb)

## Abbreviations

AOR: Adjusted odds ratio
CI: Confidence interval
GIHSN: Global Influenza Hospital Surveillance Network
IVE: Influenza vaccine effectiveness
OR: Odds ratio
RT-PCR: Reverse transcription-polymerase chain reaction
