# Supplementary material for: Influenza epidemiology and influenza vaccine effectiveness during the 2016–2017 season in the Global Influenza Hospital Surveillance Network (GIHSN)
Source: BMC Public Health. 2019 May 2;19:487. doi: 10.1186/s12889-019-6713-5 (PMC6498567; doi:10.1186/s12889-019-6713-5)
Supplement: Supplementary file 1 — Complementary Table S1. (DOCX 142 kb) [file 12889_2019_6713_MOESM1_ESM.docx]

**Complementary Table S1: Vaccines**

| **City (Country)** | **Names and types of vaccines 2016/17** | **Target population for Influenza Vaccination** |
| --- | --- | --- |
| Saint Petersburg (Russian Fed.) | The following vaccines are recommended in frame of National Calendar of vaccination for high-risk groups. They are used free of charge:  1. Grippol, MicroGene, Russia, polymer subunit inactivated vaccine with adjuvant polyoxidonium is recommended for all group of population since 6 months  2. Grippol plus, Petrovax, Russia, polymer subunit inactivated vaccine with adjuvant polyoxidonium is recommended for all groups of population since 6 months  3. Sovigripp, MicroGene, Russia, polymer-subunit inactivated vaccine with adjuvant sovidon is recommended for adults from 18 years old  4. Ultrix, FORT, Russia, split vaccine is recommended for children since 7 years old  The following vaccines are also available and registered:  5. Influvir, MicroGene, Russia, live vaccine, is recommended since 3 y.o.  6. Grippovac, Russia, inactivated virion vaccine, is recommended since 18 y.o.  7. Vaxigrip, Sanofi Aventis, France, is recommended since 6 months  8. Influvac, Abbot Biologicals, The Netherlands, is recommended since 6 months  9. Fluvaxin, Changchun Changsheng Life Sciences, China, is recommended since 6 months | Children aged 6 months and more  Students grades 1-11  Students of professional and high schools  Employees of medical and educational institutions, transport workers, service sector  Elderly > 60 years  Pregnant women  People with chronic cardiovascular and pulmonary pathology, metabolic disorders and obesity |
| Moscow (Russian Fed.) | The following vaccines are recommended in frame of National Calendar of vaccination for high-risk groups. They are used free of charge:  1. Grippol, MicroGene, Russia, polymer subunit inactivated vaccine with adjuvant polyoxidonium is recommended for all group of population since 6 months  2. Grippol plus, Petrovax, Russia, polymer subunit inactivated vaccine with adjuvant polyoxidonium is recommended for all groups of population since 6 months  3. Sovigripp, MicroGene, Russia, polymer-subunit inactivated vaccine with adjuvant sovidon is recommended for adults from 18 years old  4. Ultrix, FORT, Russia, split vaccine is recommended for children since 7 years old  The following vaccines are also available and registered:  5. Influvir, MicroGene, Russia, live vaccine, is recommended since 3 y.o.  6. Grippovac, Russia, inactivated virion vaccine, is recommended since 18 y.o.  7. Vaxigrip, Sanofi Aventis, France, is recommended since 6 months  8. Influvac, Abbot Biologicals, The Netherlands, is recommended since 6 months  9. Fluvaxin, Changchun Changsheng Life Sciences, China, is recommended since 6 months | Children from six months of age until 16 years old (school children grades 1-11)  Students of professional and vocational training institutions  Employees of medical and educational institutions, transportation, utilities  Persons older than 60 years (first of all institutionalized)  Pregnant women  Military conscription persons  Persons with chronic conditions including COPD, cardiovascular diseases, metabolic disorder, obesity, diseases and defects of central nervous system, bronchopulmonary diseases, asthma, chronic renal disease, diabetes, autoimmune disease, allergic diseases (except allergy to chicken protein), chronic anemia, congenital or acquired immunodeficiency, HIV infected. |
| Praha (Czech Republic) | Vaxigrip (Sanofi Pasteur)  Influvac (Abbott)  Optaflu (Novartis) | People above 65 years  People of any age with chronic conditions: chronic respiratory diseases; chronic cardiovascular diseases; chronic renal diseases; chronic metabolic diseases including diabetes; chronic immunodeficiency  Vaccination also recommended: pregnant women and health care and social care workers |
| Canada | TIV, QIV and LAIV vaccines are available for use in Canada. Brand names include Fluviral, Vaxigrip, Agriflu, Influvac, FluLaval, Fluzone, Fluzone high dose, Fluad, and Flumist | Influenza vaccine is recommended for everyone 6 months of age and older who do not have contraindications to the vaccine.  There are particular recommendations or a number of high risk groups including:   - All pregnant women - Adults and children with one of the following chronic health conditions:  1. cardiac or pulmonary disorders (including bronchopulmonary dysplasia, cystic fibrosis and asthma) 2. diabetes mellitus and other metabolic diseases 3. cancer, immune compromising conditions (due to underlying disease, therapy or both) 4. renal disease 5. anemia or hemoglobinopathy 6. neurologic or neurodevelopment conditions 7. morbid obesity (BMI ≥40)    - children and adolescents (age 6 months to 18 years) undergoing treatment for long periods with acetylsalicylic acid, because of the potential increase of Reye's syndrome associated with influenza    - People of any age who are residents of nursing homes and other chronic care facilities    - People ≥65 years of age    - All children 6 to 59 months of age    - Aboriginal People |
| Kazakhstan | Only TIV – Grippol plus (Petrovax), Vaxigrip (Sanofi Pasteur), Influvac (Abbot) | Health Care Practitioners, Pregnant, Children and Adult with underline chronicle conditions, children living in orphanages, elderly after 65 living in nursing houses |
| Romania | The vaccine (INFLUVAC) is provided free of charge for the recommended risk groups, being covered by the Ministry of Health budget.  The all the other brands of vaccine are also available in pharmacies. | The trivalent influenza vaccine is recommended in Romania to people with chronic diseases (i.e. respiratory, cardiovascular, renal, hepatic diseases, diabetes and metabolic disorders), HIV infected persons, pregnant women, elderly over 65 years old, institutionalized persons for social care and health care workers. |
| Istanbul Bursa Ankara (Turkey) | Vaxigrip (Sanofi Pasteur)- trivalent inactivated flu vaccine  Fluarix tetra (GSK)- quadrivalent inactivated flu vaccine (has been on the market since 2014-15 season, only 25000 doses on the market in 2016)  Fluarix (GSK)- trivalent inactivated flu vaccine (was on the market until 2015-16 season) | In Turkey, vaccination is recommended and reimbursed for elderly ≥65 years of age, people living in nursing homes and care centers for elderly, patients with chronic pulmonary diseases including asthma, patients with chronic cardiac diseases, patients with any chronic metabolic diseases including diabetes, patients with chronic renal dysfunction, adults or children with hemoglobinopathies, immune deficiency or receiving immunosuppressive treatment, adolescents and children between 6 months and 18 years old who are on long term acetylsalicylic acid treatment and health care workers. |
| Valencia (Spain) | Vaxigrip® (Sanofi Pasteur): split virus, egg based. For >6m (general population)  Intanza 15® (Sanofi Pasteur): inactivated, split. Indicated in institutionalized 60 year old or older patients. | >=60 years old, >6 months and <60 years with underlying medical conditions, persons who work with high risk people (in care home, hospitals...), institutionalized people, public services workers, pregnant women, children from 6 months to 18 years old taking aspirin |
| Srinagar, Kashmir (India) | 1. Vaxigrip (Sanofi Pasteur).  2. Influvac (Inactivated Trivalent vaccines from Abbott)  3. Biovac flu (TIV from Wockhardt, India Limited)  4. Influgen (TIV from Lupin laboratories Limited)  5. Live attenuated trivalent influenza vaccine from Serum Institute marketed by Cipla, India. | No adoption of the Influenza vaccination in the Universal Immunization Program of the Government of India. Recently (post 2015), Ministry of Health and Family Welfare has recommended influenza vaccination for health care workers and those at high risk for complications including pregnant women, young children, the elderly and those with underlying comorbidities. Other physician bodies/associations like Indian Academy of Paediatrics, Geriatric Society of India and the Federation of the Obstetricians and Gynaecologists of India have recommended vaccination for population at risk attended to by physicians working in these specialties. Data about immunogenicity and safety, however, are scant and mainly available through industry conducted studies required for generation of mandatory minimal data prior to approval of the vaccine for use on Indian subjects. Recent regulatory authorities have approved the quadrivalent influenza vaccine for use in selected age groups based on data generated from manufacturer sponsored data. Vaccine effectiveness studies do not exist for Indian subjects. Vaccine costs are borne by the individuals seeking vaccination themselves and only in some states free vaccination is provided to health care workers free of charge. Vaccination uptake is poor in all high risk individuals. |
| China |  | The target population for the influenza vaccine in China includes: 6 months -5 years old children, pregnancy woman, >60 years old adults, and people in any ages with certain chronic diseases. |
| Tunisia | Hedi Chaker University Hospital:  Influvac®  Agrippal S1®  Panenza®  Vaxigrip®  Abderrahmene Mami Ariana Hospital: Influvac | * Persons at risk of severe form or complications: Age > 65 years, people with chronic heart disease or chronic respiratory disease, mellitus diabetes, obesity.  * Health professionals.  * Pregnant woman.  * Children 2 to 5 years of age with chronic pathology.  * Any health professional in contact with subjects at risk. |
| Mexico | Fluzone and Vaxigrip (trivalent flu vaccine) – Sanofi Pasteur  Fluzone quadrivalent (quadrivalent flu vaccine) – Sanofi Pasteur  Fluarix (trivalent flu vaccine) – GlaxoSmithKline  Fluzactal Tetra (quadrivalent flu vaccine) – GlaxoSmithKline  Agrippal SI and Fluad (trivalent flu vaccine) – Novartis  Ollinflu (trivalent flu vaccine) –BIRMEX | According to our National Health Authorities, infants (above 6 months old) and children less than 5 years of age, as well as the elderly (above 60 years of age) are priority groups. Recently, vaccination has expanded to include those 5 to 59 years old at high risk for influenza (people with morbid obesity, diabetes, cardio-vascular disease, immunosuppressed, no spleen, haemoglobin disease, chronic renal disease, arthritis, during pregnancy, etc.), health workers and those caring for children (day care centres) |
| Ivory Coast | VAXIGRIP Inactivated influenza vaccine with fragmented virion. Suspension for injection in pre-filled syringe.  QUALITATIVE AND QUANTITATIVE COMPOSITION: Influenza virus (inactivated, fragmented) strains  Following: * A / California / 7/2009 (H1N1) pdm09-derived strain used (NYMC X-179A): 15 micrograms HA **;  A / Texas / 50/2012 (H3N2) -sused derivative used (NYMC X-223A): 15 micrograms HA **;  B / Massachusetts / 2/2012: 15 micrograms HA **, for a dose of 0.5 ml. * Cultivated on embryonated eggs of  Hens from healthy farms. ** Hemagglutinin. This vaccine complies with WHO recommendations  (In the Northern Hemisphere) and the decision of the European Union for the 2014/2015 season. Excipients: Solution  Buffer containing sodium chloride, disodium phosphate dihydrate, monopotassium phosphate,  Potassium chloride and water ppi. VAXIGRIP may contain traces of eggs, such as ovalbumin, traces  Neomycin, formaldehyde and octoxinol-9, used in the manufacturing process (see section "Contraindications").  PHARMACEUTICAL FORM: Suspension for injection in pre-filled syringe. The vaccine, after  Having been gently agitated, is a slightly whitish and opalescent liquid | There is no specific target group. The vaccine is administered to anyone who wishes to be vaccinated. Vaccination is not free |
| Peru | FLUQUADRIl (Sanofi’s tetravalent around 100,00 doses)  FLUARIX: Trivalent from MSD (unusually)  AFLURIA: Trivalent from CSL(unusually) | Under 5 years of age and over 65 (priority)  Health workers  Pregnant women |
| South Africa | In South Africa trivalent inactivated influenza vaccine is available. Vaxigrip has been until now the most common vaccine. | South Africa has annual recommendations for influenza vaccination since 2005, and the National Department of Health recommends that individuals at higher risk for severe disease be vaccinated against influenza, this include HIV-infected individuals and pregnant women. |
